# A genetically encoded secreted toxin potentiates synaptic NMDA receptors in hippocampal neurons and confers neuroprotection

**DOI:** 10.1093/pnasnexus/pgaf041

**Published:** 2025-02-06

**Authors:** Ido Carmi, Shaden Zoabi, Asaf M Bittan, Shai Kellner, Shimrit Oz, Ronit Heinrich, Shai Berlin

**Affiliations:** Department of Neuroscience, Ruth and Bruce Rappaport Faculty of Medicine, Technion—Israel Institute of Technology, Haifa 3525433, Israel; Department of Neuroscience, Ruth and Bruce Rappaport Faculty of Medicine, Technion—Israel Institute of Technology, Haifa 3525433, Israel; Department of Neuroscience, Ruth and Bruce Rappaport Faculty of Medicine, Technion—Israel Institute of Technology, Haifa 3525433, Israel; Department of Neuroscience, Ruth and Bruce Rappaport Faculty of Medicine, Technion—Israel Institute of Technology, Haifa 3525433, Israel; Department of Neuroscience, Ruth and Bruce Rappaport Faculty of Medicine, Technion—Israel Institute of Technology, Haifa 3525433, Israel; Department of Neuroscience, Ruth and Bruce Rappaport Faculty of Medicine, Technion—Israel Institute of Technology, Haifa 3525433, Israel; Department of Neuroscience, Ruth and Bruce Rappaport Faculty of Medicine, Technion—Israel Institute of Technology, Haifa 3525433, Israel

**Keywords:** NMDA receptors, conantokin toxins, gene delivery, pharmacogenetic tools, neuroprotection

## Abstract

NMDA receptors (NMDARs) play essential roles in neuronal development, survival, and synaptic plasticity, to name a few. However, dysregulation in receptors' activity can lead to neuronal and synaptic damage, contributing to the development of various brain pathologies. Current pharmacological treatments targeting NMDARs remain limited, for instance due to insufficient receptor selectivity and poor spatial targeting. Genetic approaches hold promise to overcome some of these issues; however, require genetically encodable NMDAR-modulating peptides, which are scarce. Here, we explored NMDAR-selective peptide toxins from marine cone snails, which resulted in the necessary engineering of a posttranslational modification-free variant of Conantokin-P (*naked* Con-P). The *naked* form is essential for expression in mammalian cells. We systematically explored the *naked* variant and discovered that *naked* Con-P maintains its ability to inhibit GluN2B-containing receptors, but uniquely acquired the ability to potentiate GluN2A-containing synaptic receptors. We then engineered a secreted *naked* Con-P that readily enhances NMDAR-mediated synaptic events in primary hippocampal neurons, and mitigates neuronal damage induced by staurosporine. We therefore provide a genetically encodable, subtype selective, and secreted bimodulator of NMDARs. This new variant and approach should pave the way for the development of additional genetic tools, specifically tailored to target NMDARs within distinct cellular populations in the brain.

**Significance Statement**

NMDA receptors (NMDARs) are crucial glutamate receptors in the brain, fundamental to neuronal development and plasticity, thereby closely associated with various neurological diseases. This study introduces a unique strategy for selectively targeting and modulating the activity of NMDAR subtypes. By altering the Conantokin-P toxin from cone snails, we developed a Con-P variant (*naked*) that enhances GluN2A-containing receptors, while inhibiting GluN2B-containing receptors. Collectively, *naked* Con-P can enhance synaptic events and protect primary hippocampal neurons from damage. We then engineer a secreted *naked* Con-P that is expressed by neurons and proffers neuroprotection. This approach offers a promising direction for engineering genetic tools to treat brain disorders linked to NMDAR dysfunction.

## Introduction

The *N*-methyl-D-aspartate receptor (NMDAR) is an essential glutamatergic receptor located mostly in excitatory synapses, where it plays pivotal roles in synaptic plasticity, neuronal survival, and development (1). NMDARs are predominantly heterotetramers composed of two obligatory GluN1 glycine-binding subunits combined with two GluN2 glutamate-binding subunits (generated from four different genes; *GRIN2A-D*) (1). This yields a large palette of possible receptor subtypes, each with its own biophysical properties, expression pattern, cellular localizations, and specializations within neurons. Paradoxically, aside from their pivotal roles in neuronal survival, NMDARs are also excitotoxic (2).

The list of brain pathologies associated with NMDARs is quite extensive, including ischemic stroke, epilepsy, and many neurodegenerative diseases, notably Alzheimer's disease (AD) (3–8). In this context, it is emerging that NMDAR hyperactivation plays a central role in cellular excitotoxicity by allowing excessive calcium influx into cells, in particular via GluN2B-containing receptors (GluN2Bs in brief) located outside the synapse (extrasynaptic) (3, 9, 10). In contrast, activation of synaptic NMDARs, mainly consisting of GluN2As in the adult brain, promotes neuroprotection (4, 5, 9, 10). Unfortunately, the current toolbox of pharmacophores is lacking modulators that can target (whether positively or negatively) select NMDAR subtypes in defined brain regions.

We envisioned that these limitations may be addressed by means of pharmacogenetic “tools,” namely protein modulators that can be expressed in defined cellular populations and at select regions of the brain (6). These tools typically consist of membrane-anchored peptide toxins derived from the venoms of predatory animals (7, 8, 11). When expressed in neurons, the membrane-tethered toxins (t-toxins) bind to and inhibit (or block) their target receptors or channels, as we have recently engineered toward a potassium channel (12). Currently, there are approximately a dozen tools to selectively inhibit various neurotransmitter receptors and voltage-gated channels. However, the development of genetically encoded modulators for NMDARs presents a significant challenge in comparison with other receptors, owing to the limited number of NMDAR-selective peptides identified to date (13–15).

The palette of peptide toxins targeting NMDARs is very scarce, with only one family of peptides exhibiting unique selectivity toward NMDAR subtypes, denoted Conantokin toxins (Cons). Cons are small toxins (15–30 amino acids) found in the venoms of the *Conus* family of sea snails (16). There are approximately a dozen variants with submicromolar affinities toward different NMDAR subunits, although the majority exhibit a surprising preference toward the inhibition of GluN2B-containing receptors (17). We deemed these features (i.e. selectivity and inhibition of GluN2Bs) beneficial for our aim, as GluN2Bs are the most implicated in excitotoxicity, synaptic damage, and neuronal death. In fact, Cons have been considered as therapeutic tools previously (18, 19); however, none of the various Cons have been genetically encoded. This results from the necessity of Cons to undergo unique chemical posttranslational modifications in the snail to convert glutamate residues within the toxin to gamma-carboxyglutamate (Gla). Gla are suggested to be essential for stabilizing the structure and thereby the function of the toxin (Table 1) (20, 21). Nevertheless, this process cannot be carried out in most mammalian cells, notably neurons. Thus, whether, and to what extent, Gla residues are essential for the activity of different Cons remains unclear.

**Table 1.** Peptide sequences of Con-G and Con-P and their respective synthetic canonical derivatives.

	Con-G	Con-P
Native toxin	GEγγLQγNQγLIRγKSN	GEγγHSKYQγ**C**LRγIRVNKVQQγ**C**
Naked toxin	GEEELQENQELIREKSN	GEEEHSKYQE**C**LREIRVNKVQQE**C**
Scrambled toxin	—	IQQVL**C**KER**C**RVEEENQSYGEKHE

γ indicates glutamate residues with a gammacarboxyl group (Gla residues). Replacement of modified residues by the canonical glutamate (E) residue in the synthetic peptides is indicated. Cysteines are highlighted (bold).

Here, we have produced and systematically scrutinized the activity of two GluN2B-selective Cons: Con-G and Con-P, after these have been stripped of their Gla residues, denoted *naked* Con-G and *naked* Con-P. We find that purified *naked* variants remain functional as inhibitors of GluN2Bs, although with reduced potency, efficacy, and selectivity. Importantly, *naked* Con-P exclusively gained the ability to potentiate GluN2As. We envisioned that the dual capacity of *naked* Con-P could be harnessed for the concomitant inhibition of GluN2Bs (implicated in apoptosis (22, 23)) and potentiation of GluN2As (implicated in neuronal survival (4, 5, 9, 10)) for providing neuroprotection. Below, we provide evidence demonstrating the neuroprotective effect of a secreted *naked* Con-P variant on hippocampal neurons.

## Results

### Characterization of purified *naked* Cons

To assess the feasibility of encoding Cons within neurons, we focused on native Cons with a natural preference for inhibiting GluN2Bs. Specifically, we selected Con-G (derived from *Conus geographus*) and Con-P (*Conus purpurascens*), as Con-G is the most extensively studied variant and, as previously mentioned, has been suggested to retain functionality in its *naked* form, albeit at a moderate level (19). Moreover, Con-P, though much less studied (and the role of its Gla residue has yet to be explored), diverges from all other Cons by including two cysteines in its sequence; suggested to form a disulfide bond (Table 1, cysteines in bold). We hypothesized that the disulfide bond may allow the peptide to retain its structure—and function—even without the Gla residues. Indeed, the helical structure of this peptide was shown to be largely unaffected when the Gla residues were not engaged (i.e. in the absence of divalent cations that bind to Gla residues for maintaining the structure of the peptide) (24).

**Fig. 1.**
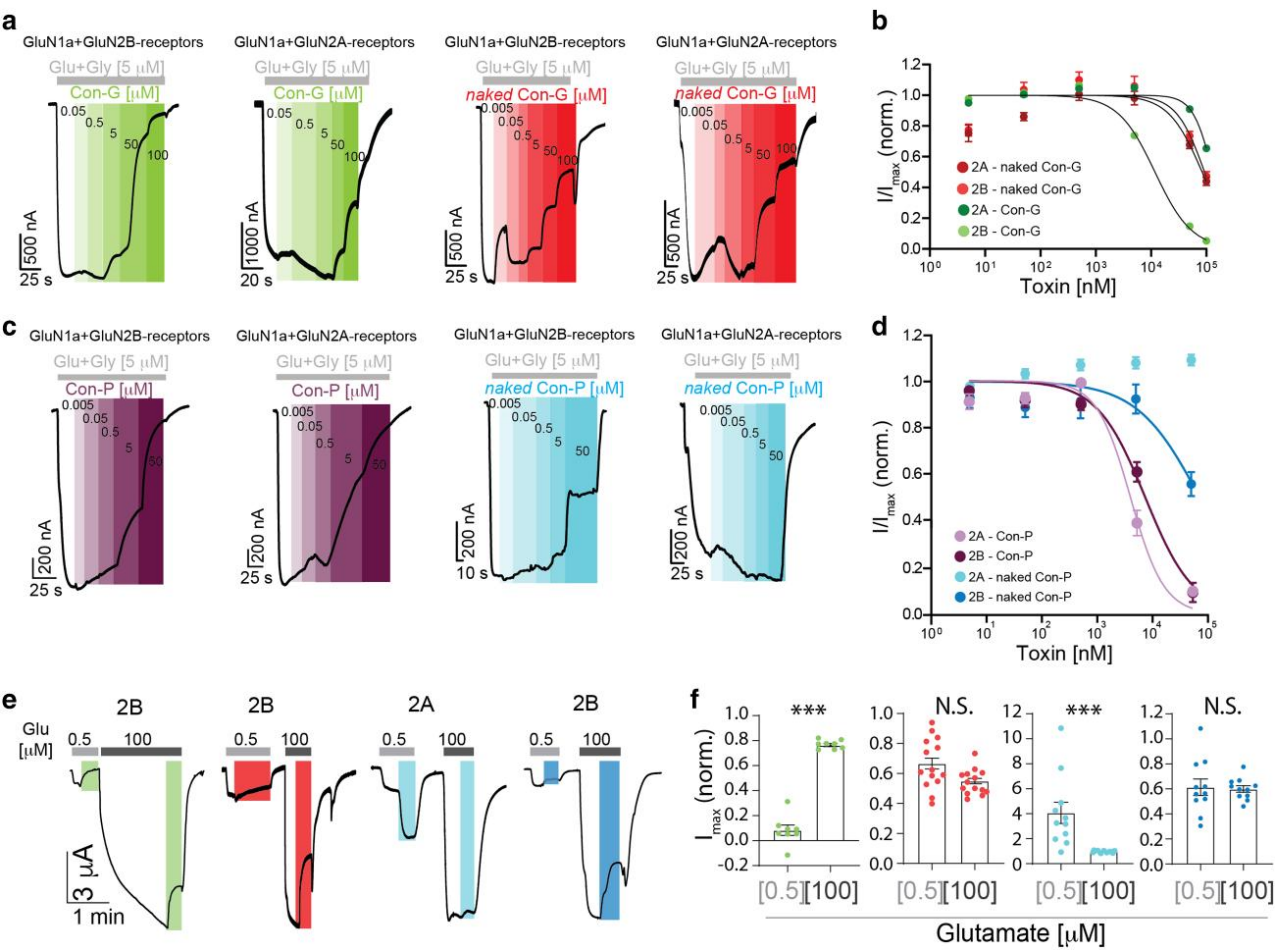
Pharmacological characterization of native and *naked* Cons over NMDARs in *Xenopus* oocytes. a) Representative traces from *Xenopus* oocytes expressing GluN2B- or GluN2A-containing receptors following continuous application of 5 μM glycine and glutamate (gray bars) and incrementing concentrations of native Con-G (left traces), *naked* Con-G (right traces). Color gradients indicate increasing toxin concentrations; summarized in (b). b) Dose–response curves of native and *naked* Con-G toxins over GluN2A or GluN2B receptors. c) Representative traces from *Xenopus* oocytes expressing GluN2B- and GluN2A-containing receptors following continuous application of glycine and glutamate and incrementing concentrations of Con-P (left traces) or *naked* Con-P (right traces). Summarized in (d). d) Dose–response curves for native Con-P and *naked* Con-P. e) Competitiveness of toxin variants. Traces show the effect of a single toxin concentration (native Con-G and *naked* Con-G = 100 μM; *naked* Con-P = 50 μM) over GluN2A-containing (middle traces) or GluN2B-containing receptors (left and right traces) activated by subsaturating (0.5 μM) or saturating (100 μM) agonist concentrations, summarized in (f). Sigmoidal fits were generated by adapted Hill equation (see Materials and methods), and statistical significance was tested by paired t tests. N.S., nonsignificant; ***P < 0.001.

We produced purified native- and *naked* Con-G and Con-P peptides and probed their modulatory activity over GluN2A- and GluN2B-containing receptors expressed in *Xenopus* oocytes (see Materials and methods). We assessed IC_50_ for Con-G as previously done by others (25), specifically, we and others do not employ saturating glutamate concentrations (e.g. 100 μM), as Con-G is an established competitive antagonist of GluN2Bs. Importantly, the agonist concentrations employed are compatible with estimations of physiological synaptic glutamate release (26). Control experiments showed that native Con-G is indeed a potent inhibitor of GluN2Bs, with minimal effect over GluN2As (Fig. 1a, left panels, and summary in Fig. 1b). Interestingly, *naked* Con-G peptide displayed a parabolic behavior toward the inhibition of both GluN2B and GluN2A receptors, namely stronger inhibition at low concentrations of the toxin followed by a decline and, lastly, incline in inhibition at higher concentrations (Fig. 1a and b; red). These prevented accurate fitting of the data for extracting IC_50_ (see Materials and methods), which was further exacerbated by the limited solubility of *naked* Con-G (∼100 μM) and thus failure to reach full inhibition. However, at the maximal soluble concentration (50–100 μM), inhibition by the toxins neared 50% of the currents, which is suitable to represent IC_50_ (Table 2). This reasoning was employed in previous studies exploring other Con-G variants, in which similar peculiar effects were observed (27). Thus, the absence of Gla residues reduced Con-G's potency and selectivity, enabling it to similarly inhibit both GluN2A and GluN2B. This combined inhibitory action has not been previously considered, and thereby likely underlies the stronger inhibition observed in neurons by another Con-G variant, in contrary to the authors' conclusion (19).

**Table 2.** Pharmacology of toxin variants over NMDAR subtypes.

Toxin	Receptor subtype	Glutamate (μM), *n*			
		0	0.5	5	100
Con-G (100 μM) (% from *I*_max_)	GluN2A			65.5 ± 2.02 (12)	
	GluN2B		8.31 ± 4.14 (8)	4.69 ± 0.44 (21)	76.36 ± 1.24 (8)
*Naked* Con-G (100 μM) (% from I_max_)	GluN2A			43.83 ± 2.48 (oocytes, 21) 72.81 ± 1.43 (HEK293T, 3)	
	GluN2B		66.59 ± 3.59 (14)	47.35 ± 2.9 (oocytes, 30) 67.7 ± 5.96 (HEK293T, 5)	54.94 ± 1.79 (14)
*Naked* Con-P [50 μM] (% from I_max_)	GluN2A	0.04 ± 0.01 (12)	340.57 ± 64.05 (10)	109.3 ± 0.59 (oocytes, 16) 155.51 ± 12.76 (HEK293T, 14)	96.8 ± 2.08 (10)
	GluN2B		61.37 ± 6.6 (11)	64.74 ± 5.82 (oocytes, 10) 80.38 ± 3.24 (HEK293T, 15)	59.94 ± 2.6 (11)
	1/2A*/2B*			71.42 ± 0.7 (7)	
	GluN2C			99.4 ± 0.93 (12)	
	GluN2D			77.77 ± 0.84 (18)	
	2A (N-2B)			72.64 ± 0.85 (7)	
	2B (N-2A)			68.25 ± 0.81 (6)	

Modulation of glutamate-evoked NMDAR responses in oocytes and HEK293T cells by Con-G, *naked* Con-G and *naked* Con-P. Responses are presented as mean ± SEM (*n*; number of cells).

In parallel, we find that native Con-P attains strong maximal inhibition of both receptor subtypes, with similar IC_50_ values (Fig. 1c and d; purple), whereas *naked* Con-P exhibited reduced potency toward GluN2Bs (Fig. 1b, d and Table 2); however, and quite unexpectedly, potentiated GluN2As (+∼9%), reaching maximal effect past 10 μM toxin (Fig. 1c, cyan, and d). Potentiation was even more pronounced at low glutamate concentrations (0.5 μM), albeit abolished by saturating glutamate concentrations (100 μM; Fig. 1e and f; cyan). Specifically, 50 μM *naked* Con-P induced a 2-fold potentiation of the current when the GluN2As were evoked by 0.5 μM glutamate concentration (Fig. S1a). Notably, *naked* Con-P was the only toxin derivative that showed this unique potentiating effect over NMDARs among all peptides examined in this work (Fig. S1b). These effects have also been observed for the Con-G variants, whereby the inhibition of the native toxin is strongly attenuated by high glutamate concentrations, but the inhibition by the *naked* Con-G is not (Fig. 1e and f, red; see summary in Table 2). Thus, the inhibition of GluN2Bs by *naked* Con-G and Con-P is not competitive, as that of the native Con-G toxin (25). Nevertheless, the reduction in the extent of potentiation specifically by *naked* Con-P over GluN2As can be interpreted as that of a partial agonist or of a positive allosteric modulators (PAMs) (28–30).

To probe for partial agonism and other features that may affect GluN2As' current, we directly applied the maximal concentration of *naked* Con-P onto oocytes expressing GluN2As, after these have been exposed to glycine only. The application of glycine did not yield any observable NMDAR responses (Fig. S1c). We also explored potential contaminations in solutions that may yield partial agonism, by conducting the experiments in 10 mM tricine buffer to chelate zinc (a potent inhibitor of GluN2As; in nM (31–33)). Remarkably, *naked* Con-P induced even larger potentiation (∼2-fold) of GluN2As, when the receptors were disinhibited by nominal zinc (+∼16% at 50 µM; Fig. S2a). We also concluded that potentiation was not due to extended recording times, which typically induces mild desensitization instead (Fig. S2b). Lastly, we explored the effect of a sequence-scrambled *naked* Con-P peptide (Table 1, scrambled). Strikingly, *scrambled* Con-P produced the exact opposite effect over GluN2As (Fig. S1d), namely dose-dependent inhibition (Fig. S2c). Though unexpected, this result is supported by multiple reports describing inhibition of NMDARs by short peptides that do not necessarily contain any specific binding signatures (e.g. (34, 35), and see below). These result from the highly “sticky” nature of NMDARs, which are shown to bind a wide range of endogenous and exogenous peptides promiscuously (reviewed in Ref. (36)). Nevertheless, in all of these and other reported instances, inhibition is always observed, whereas here we describe the first instance of selective potentiation. Next, to exclude the possibility that the selective potentiation of *naked* Con-P stemmed from noncanonical NMDARs that may be present in the oocytes (37), namely endogenous *Leavis* NMDAR subunits that could co-assemble with the exogenous GluN1a, we expressed this subunit alone in various oocyte batches and probed for NMDAR current. We find no indication for NMDAR currents (Fig. S3), supporting recent refutes of this matter (38). Lastly, to rule out the possibility that the unique potentiating behavior of *naked* Con-P was caused by unknown endogenous factors that might be expressed within the oocyte expression system, we repeated the dose response for *naked* Con-G and *naked* Con-P in mammalian Human Embryonic Kidney cells (HEK293T) in which we co-expressed GluN1a with GluN2A or GluN2Bs (Fig. S4), and recapitulate all the results obtained in the oocytes. Specifically, *naked* Con-G induced inhibition of both GluN2As and GluN2Bs, on par with the effect observed in oocytes (Table 2). Similarly, *naked* Con-P inhibited GluN2Bs, albeit to a lesser extent (∼20% maximal inhibition) and—importantly—potentiated GluN2A-containing receptors to an even larger extent (+55%; Fig. S4b).

### *Naked* Con-P's selectivity and mechanism

To further explore the selectivity of naked Con-P, we examined their effect over GluN2C- and GluN2D-containing diheteromers, as well as over triheteromeric receptors composed of GluN1a/GluN2A/GluN2B receptors (highly abundant in adult brain). We found no significant effect over GluN2Cs, but we do see minimal inhibition of GluN2Ds (Fig. S5a and b; Table 2), as described for the native Con-P (24). To examine the effect of *naked* Con-P over GluN1a/GluN2A/GluN2B triheteromers, we used an endoplasmic reticulum retention technique for selective expression of recombinant NMDARs (see Materials and methods) (39, 40). We confirmed that triheteromeric currents of 1a/2A-C1/2B-C2 were 23- and >100-fold larger than leak currents obtained from 1a/2A-C1/2A-C1 or 1a/2B-C2/2B-C2 diheteromers, respectively (Fig. S5c) and that the application of 50 μM of *naked* Con-P exerted inhibition (∼25%) of the channels (Fig. S5d and e).

To gain insights into the mechanism by which *naked* Con-P potentiates GluN2As, we examined chimeric NMDARs in which the amino-terminal domains (ATDs, or N in brief) were swapped between the two subunits, yielding a GluN2A subunit with the “N” of GluN2B (2A-(N-2B)) and inversely 2B-(N-2A) (41). We chose this region of the receptor as it contains most of the known modulatory binding sites (1, 29, 30). Indeed, this notion is supported by a previous report examining an alanine-substituted Con-G, in which instance the variant competed with the binding of spermine (42). We find that *naked* Con-P minimally, albeit significantly, inhibited whole-cell currents of 2A-(N-2B) by 28% (instead of the expected +∼55% potentiation of GluN2As), however, maintained the extent of inhibition (−32%) over 2B-(N-2A) (Fig. S5f). These suggest that the potentiating effect of *naked* Con-P over GluN2A-containing receptor likely involves a concerted structural dependence between the ATD and the LBD, supported by the observation that GluN2B-(N-2A) did not undergo potentiation by the peptide, instead inhibition was strictly preserved (Fig. S5g; Table 2).

Finally, we explored the effect of the peptide over various synaptic receptors that either share sequence homology with NMDARs or that may directly modulate synaptic NMDAR activity. These include the principal glutamatergic α-amino-3-hydroxy-5-methyl-4-isoxazolepropionic-acid receptor (AMPAR), the metabotropic gamma-aminobutyric-acid type b receptor (GABA_B_R) and the mGluR1- or mGluR2-containing metabotropic glutamate receptors (43–46). In the case of the metabotropic GABA_B_ and mGluR receptors, we measured receptor activation by co-expression of the G protein-coupled inwardly rectifying potassium channel (GIRK1/2), whereas recorded endogenous Ca^2+^-activated chloride currents when exploring mGluR1 (47). In all cases, *naked* Con-P had no significant effect over these receptors and channels (Fig. S6).

### *Naked* Con-P differentially modulates NMDAR-mediated postsynaptic activity in young and mature hippocampal neurons

**Fig. 2.**
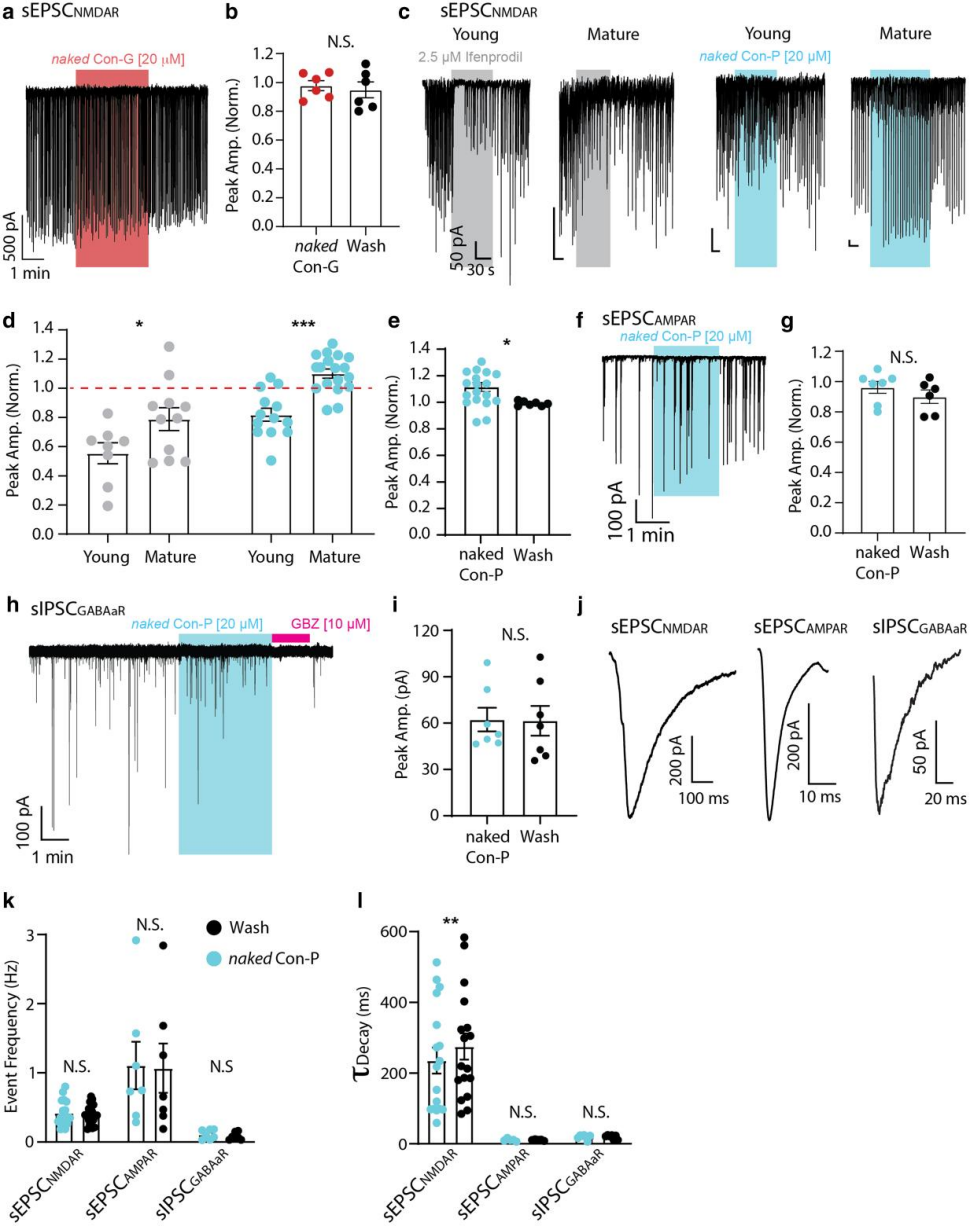
*Naked Con-P* enhances NMDAR-dependent synaptic activity in hippocampal neurons. a) Representative whole-cell recordings from a hippocampal neuron showing NMDAR-mediated spontaneous EPSCs (sEPSC_NMDAR_) before (wash) and following the application of 20 μM *naked* Con-G (bar); summarized in b). sEPSC_NMDAR_ were isolated by 20 μM CNQX and 10 μM GBZ. Statistical significance tested by paired t test. c) Representative whole-cell voltage-clamp recordings of sEPSC_NMDAR_ from young or mature neuronal cultures, following the application of 3 μM Ifenprodil (2 left bars) or 20 μM *naked* Con-P (2 right bars); summarized in (d). Dashed red line indicates baseline sEPSC_NMDAR_ amplitude for each group (during wash). Significance of repeated measures tested by two-way ANOVA, followed by post hoc Tukey test. e) *Naked* Con-P enhances peak amplitude of sEPSC_NMDAR_ (compared with baseline; wash); statistical significance tested by paired t test. f–i) *Naked* Con-P has no effect over AMPA- or GABA_a_ receptors. Representative whole-cell recordings from mature hippocampal neurons showing AMPAR-mediated spontaneous EPSCs (sEPSC_AMPAR_) (f) or GABA_a_R-mediated sIPSCs (h), before and after the application of 20 μM *naked* Con-P (bars); summarized in (g) and (i), respectively. sEPSC_AMPAR_s were isolated by the application of 20 μM AP5 and 10 μM GBZ. sIPSC_GABAaR_ were isolated by 20 μM AP5 and 20 μM CNQX. sIPSCs_GABAAR_ were further confirmed by the application of gabazine (GBZ [10 μM]). Statistical significance tested by paired t tests. j) Averaged NMDAR− (left), AMPAR− (middle), or GABA_a_R− (right) mediated postsynaptic events from three different mature hippocampal neurons in the presence of the appropriate channel blockers (20 μM CNQX/20 μM AP5/10 μM GBZ). k, l) Summaries of the effect of 20 μM *naked* Con-P over frequency and deactivation kinetics (*τ*_deact._) of NMDAR-, AMPAR-, and GABA_a_R-mediated postsynaptic events. Statistical significance of repeated measures was tested by two-way ANOVA, followed by post hoc Tukey test. N.S., nonsignificant; *P < 0.05; **P < 0.01; ***P < 0.001.

We proceeded to test the toxins' effect over endogenous NMDARs in synapses of hippocampal neurons. We isolated spontaneous NMDAR-dependent excitatory postsynaptic currents (sEPSC_NMDAR_) by holding neurons at −70 mV and perfused with a cocktail of blockers against AMPARs (20 μM CNQX) and GABA_A_ receptors (10 μM Gabazine; GBZ); before and after the application of the toxins at subsaturating concentrations (19). We deliberately employed 20 μM *naked* Con-G and *naked* Con-P (instead of 50–100 μM for brief applications onto HEK cells and oocytes) to avoid nearing the solubility limit of the peptides and prevent precipitation and fluctuations in peptide concentrations. Importantly, at this concentration, the effects of the peptides near (∼90%) the maximal effect obtained in oocytes and HEK cells (see Figs. 1 and S4). Under these settings, *naked* Con-G did not induce any observable modulation of sEPSC_NMDAR_'s amplitude (Fig. 2a and b). To explore the dual capabilities of *naked* Con-P in endogenous receptors subtypes in hippocampal neurons, we compared sEPSC_NMDAR_ from young (7–10 days in vitro, DIV) to those of mature (15–21 DIV) neuronal cultures. Briefly, GluN2Bs are suggested to be the predominant synaptic receptor subtype in young neurons, and as neurons mature, a GluN2B-to-GluN2A swap occurs, leading to an excess of synaptic GluN2As over GluN2Bs (1). We validated this by use of ifenprodil (at 2.5 μM) in which instance it strongly reduced amplitudes of sEPSC_NMDAR_ in young, but significantly less so in mature neurons (Fig. 2c and d; left pannels and bars, respectively). Then, the application of *naked* Con-P onto young neurons instigated a rapid and significant reduction in sEPSC_NMDAR_'s amplitude, and this effect was reversed in mature neurons, in which we observed robust increases in amplitude (Fig. 2c and d; right panels and bars, respectively). This increase is further supported by the increase in the mean amplitude of sEPSC_NMDAR_ in mature neurons compared with the mean of age-matched neurons that were not exposed to the drug (Fig. 2e). We further recorded AMPAR-mediated sEPSCs (sEPSC_AMPAR_) or GABA_a_R-mediated sIPSCs (sIPSC_GABAaR_) in separate sets of neurons, before and after the application of 20 μM *naked* Con-P (sEPSC_AMPAR_ isolated via 20 μM (2R)-amino-5-phosphonovaleric acid [AP5] and 10 μM GBZ; sIPSC_GABAaR_ by a high-chloride intracellular solution and application of 20 μM AP5 and CNXQ) (48). We found no modulation of the amplitude of sEPSC_AMPAR_ by *naked* Con-P (Fig. 2f and g), as well as no effect over sIPSC_GABAaR_ peak amplitude (Fig. 2h and i). We compared the deactivation kinetics (*τ*_decay_) in which instances 20 μM *naked* Con-P had no effect over the frequency or kinetics of AMPAR and GABA_a_R; however, it did instigate a mild, albeit significant, reduction in deactivation kinetics of sEPSC_NMDAR_s (Fig. 2j–l; Table 3). This suggests a larger fraction of current being evoked by postsynaptic GluN2As in the presence of *naked* Con-P. Thus, *naked* Con-P remains efficacious in synapses and differentially modulates neuronal NMDAR GluN2A and GluN2B subtypes, without an effect over non-NMDAR glutamatergic and GABAergic activities.

**Table 3.** *Naked* Con-P effects over frequencies and decay times of postsynaptic events by receptor type.

	Event frequency (Hz)			*τ*_decay_ (ms)		
	Wash	*Naked* Con-P	*n*; *P*-value	Wash	*Naked* Con-P	*n*; -value
sEPSC_NMDAR_	0.39 ± 0.03	0.41 ± 0.04	19; 0.23	275.4 ± 37.21	235.6 ± 36.85	19; < 0.01
sEPSC_AMPAR_	1.07 ± 0.35	1.1 ± 0.34	7; 0.51	10.93 ± 0.75	10.81 ± 1.68	7; 0.94
sIPSC_GABAaR_	0.08 ± 0.03	0.11 ± 0.04	7; 0.22	19.49 ± 2.34	19.87 ± 2.42	7; 0.88

Responses are presented by mean ± SEM (*n*; number of cells) from mature hippocampal neurons before (wash) or during the application of 20 μM *naked* Con-P.

As NMDARs are the principal source of postsynaptic calcium conductance (1), we validated the dichotomous effect of *naked* Con-P over young and mature neurons by examining dendritic calcium activity. We postulated that *naked* Con-P should reduce calcium in young (2A<<2B), and the inverse in mature neurons (2A>>2B). We thereby expressed GCaMP7f (49, 50) in these neurons and recorded calcium activity before and during the application of native or *naked* Con-P (Fig. S7a and b). We observed that native Con-P strongly suppressed dendritic activity in young neurons, and this inhibition was significantly attenuated in mature cultures (Fig. S7c and d, top panels). Conversely, *naked* Con-P did not affect the calcium transients in young cultures, however significantly increased the latter in mature cultures (Fig. S7c and d, bottom panels). These are highly consistent with the electrophysiological recordings (above).

### *Naked* Con-P attenuates staurosporine-induced apoptosis in mature, but not young, cultured hippocampal neurons

Potentiation of NMDAR-mediated synaptic currents in mature neurons promotes prosurvival mechanisms, along with downregulation of apoptosis and reduction of neuronal death following various cytotoxic insults (51, 52). One such established insult is induced by staurosporine (STRO). STRO, a protein kinase C inhibitor, reduces the efficacy of NMDARs activity at the synapse and promotes apoptosis (53). Importantly, pharmacological increases in synaptic NMDAR-dependent activity prior to STRO application rescues neuronal degeneration (9). We therefore hypothesized that potentiation of GluN2A-mediated synaptic activity by *naked* Con-P could rescue neurons following STRO. We exposed young and mature neurons to 500 nM *STRO* for 48 h, with or without 24 h pretreatment with 20 μM *naked* Con-P (Fig. S8). Neurons were then fixed and stained with Hoechst 33342 and NeuroTrace to track STRO-induced DNA breakage specifically in neurons (Fig. S8a). Control neurons (non-STRO treated) from young and mature cultures did not show any apoptotic neurons, whereas STRO-induced apoptosis in approximately one-third (30.85%) of young neurons (Fig. S8b). The extent of STRO-induced degeneration was similarly observed in young cultures that were pretreated with *naked* Con-P (Fig. S8b, cyan). Therefore, inhibition of GluN2Bs alone is insufficient to rescue young neurons from STRO-induced death. However, we observed a marked neuroprotective effect following preincubation of mature neurons with *naked* Con-P, in which *naked* Con-P preincubation strongly attenuated apoptosis (from 76.99% dead neurons to 46.45% of neurons). Co-application of AP5 and *naked* Con-P abolished the toxin's neuroprotective effect, suggesting a specific NMDAR-mediated effect of *naked* Con-P. In fact, preincubation with AP5 alone (before STRO application) did not exert neuroprotection (Fig. S8b). These results imply that broad (e.g. via AP5) inhibition of NMDARs does not protect against damage by STRO, supporting previous observations showing that neuroprotection is obtained specifically by activating GluN2As (9). Here, we show that potentiation yields similar neuroprotection, thereby highlighting the potential of using PAMs for synaptic NMDARs to promote neuronal survivability.

To test the specificity of the neuroprotective effect of *naked* Con-P, we conducted another survivability experiment in which neurons were exposed to NMDA excitotoxicity (Fig. S8c). Briefly, exposure of neurons to excessive NMDA results in neuronal damage likely due to activation of extrasynaptic GluN2Bs (9, 22). We therefore postulated that the pretreatment of neurons with *naked* Con-P should not exert a neuroprotective effect, owing to its relatively weak effect over GluN2Bs. We tracked neurons for 48 h by continuous live imaging (via Incucyte; see Materials and methods) and counted dead neurons (neurons that have progressively lost their morphology, where cell debris was visible or neurons that have disappeared) (9). NMDA application induced robust excitotoxicity of mock tdTomato-infected neurons (killing ∼80% of the neurons), whereas non-NMDA treated remained highly viable (~95% alive). Pretreatment with 20 μM *naked* Con-P of hippocampal neurons did not rescue these from the insult (Fig. S8c and d). Thus, in contrast to its neuroprotective effect against STRO, *naked* Con-P did not protect against NMDA excitotoxicity.

### A genetically encoded secreted version of *naked* Con-P enhances synaptic NMDAR activity in hippocampal neurons

We next aimed to produce a genetically encoded tool to modulate endogenous NMDARs, such as membrane-tethered peptides (see Introduction) (8, 11, 12). We designed a membrane-tethered *naked* Con-G and Con-P consisting of a signal peptide (SP), followed by the sequence of the toxins, a transmembrane domain (PDGFR) and a tdTomato for visualization. We expressed GluN2Bs in HEK293T cells and compared whole-cell currents between cells with or without co-expression of the t-toxins. Unfortunately, and despite robust expression and localization of the t-toxins at the plasma membrane (Fig. S9a and b), whole-cell NMDAR currents were not affected by the presence of t-Con-G or t-Con-P compared with control conditions (a membrane-tethered clone lacking the toxin or with a potassium channel tethered heteropoda toxin, denoted MetaPoda (12); Fig. S9c–f). In hindsight, we should have expected this strategy to fail since the tethered-toxin approach, while proven efficacious for inhibiting (even ultrapotently (12)) many other channel types, is limited by the affinity of the toxin toward its target receptor (11) as the effective concentration of t-toxin at the membrane surface is estimated to reach submicromolar concentrations, and much higher concentrations are needed for *naked* Con-P to have an effect over NMDARs. Thus, to be functional, t-toxins should exhibit higher (nanomolar) affinity toward their target (54).

**Fig. 3.**
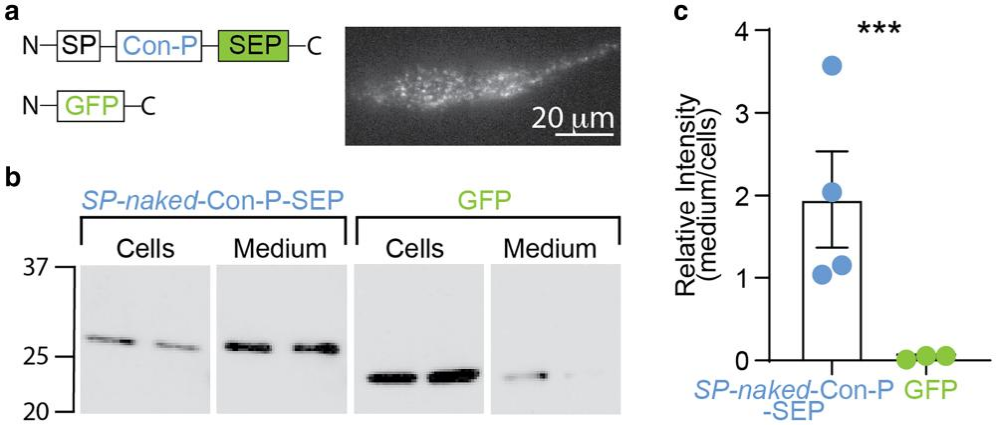
A genetically encoded secreted *naked* Con-P variant is efficiently secreted from differentiated PC-12 cells. a) Illustration of a secreted form of *naked* Con-P tagged with a pH-sensitive GFP (supereclyptic pHluorin, SEP) and a control construct consisting of the fluorescent protein alone (GFP). Secretion is obtained by the inclusion of BDNF's SP upstream of *naked* Con-P (see Fig. S10). (Inset) Expression of the SP-*naked*-Con-P in secretory vesicles in differentiated PC-12 cells visualized by TIRF microscopy. b) Presence of SP-*naked*-Con-P and GFP were detected in culture media or within cells (lysates) by western blotting against GFP (*n* = 2). c) Quantitation of the amount of secreted peptide, by ratioing band intensities (amount) of peptides in media compared with its amounts within cells (cells) from four independent experiments. Statistical significance tested by unpaired t test. ***P < 0.001.

**Fig. 4.**
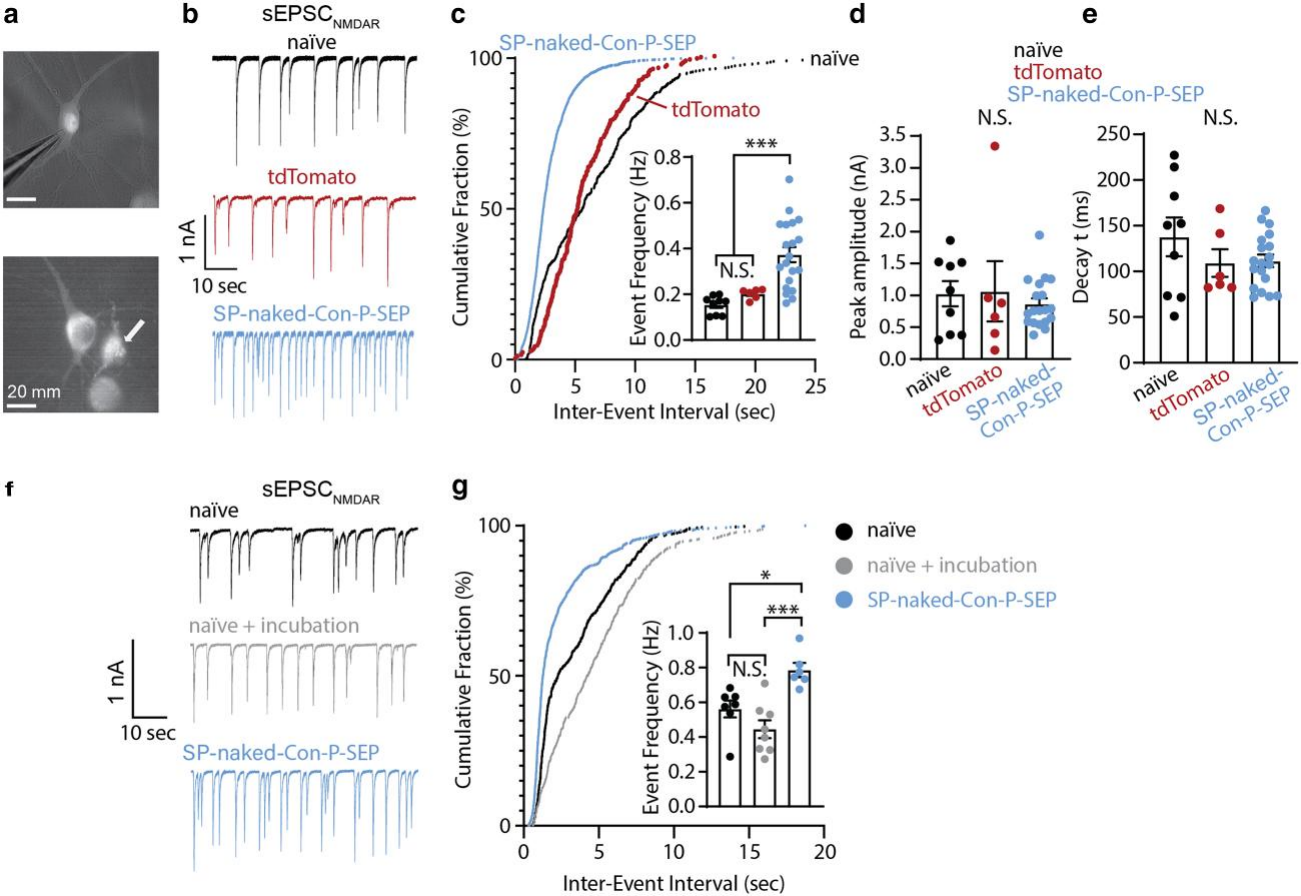
Secreted *naked* Con-P-SEP facilitates NMDAR-mediated sEPSCs in hippocampal neurons. a) Images of hippocampal neurons expressing tdTomato (top) or *naked* Con-P-SEP (bottom) following viral infection. Secretory vesicles containing *naked* Con-P-SEP can be observed in infected neurons (bottom panel, arrow). Scale bar = 20 μm. b) Representative whole-cell recordings of sEPSC_NMDAR_ from noninfected neurons (naïve, top trace) compared with mock-infected neurons (virally infected to express tdTomato, middle trace) or infected with AAV1 to express SP-*naked*-Con-P-SEP (bottom trace). sEPSC_NMDAR_ were isolated by using 20 μM CNQX and 10 μM GBZ. c) Cumulative distributions for sEPSC_NMDAR_ interevent intervals for the three different groups in b) (Inset) Effect of SP-*naked*-Con-P-SEP over mean sEPSC_NMDAR_ event frequency. d, e) Summaries of sEPSC_NMDAR_'s amplitudes and deactivation kinetics, respectively. f) Representative whole-cell recordings of sEPSC_NMDAR_ from naïve neurons (top trace), noninfected neurons that were incubated (48 h) with enriched media taken from SP-naked-Con-P-SEP expressing cultures (middle trace), and neurons virally expressing SP-*naked*-Con-P-SEP (bottom trace). g) Cumulative distributions for sEPSC_NMDAR_ interevent intervals for groups in (f). (Inset) Effect of SP-*naked*-Con-P-SEP over mean sEPSC_NMDAR_ event frequency. Statistical significance tested by one-way ANOVA followed by post hoc Tukey analysis. N.S., nonsignificant; *P < 0.05; ***P < 0.001.

We therefore pursued an alternative approach for genetically encoding *naked* Con-P to target synaptic receptors. We focused on the secretory pathway by which neurons release proteins to the extracellular space, such as the brain-derived neurotrophic factor (BDNF). In neurons, BDNF is secreted to the synaptic cleft, where it binds to TrkB-tyrosine kinase receptor and mediates neuronal survival and differentiation, promotes the formation of new synapses (55–57), and facilitates synaptic NMDAR activity (58, 59). To leverage this secretory route, we designed clones in which we introduced elements from the BDNF protein for obtaining secretion. In the first, the sequence of *naked* Con-P was preceded by the SP of BDNF, as well as tagged with a pH-sensitive superecliptic green fluorescent protein (SEP) (60) at its carboxy-terminal (denoted SP-*naked*-Con-P-SEP), whereas the second clone included the entire preprocessed BDNF sequence (SP-PreProPeptide-*naked*-Con-P-SEP; Fig. S10a). As controls, we employed an intracellular GFP or a secreted BDNF clone. We tested the clones in differentiated PC-12 cells, a model system for vesicle secretion (61). Briefly, after 5 days of differentiation by neuronal growth factor (NGF) supplementation, we transfected cultures with BDNF-SEP, SP-*naked*-Con-P-SEP or PrePro-Con-P-SEP, then after 3 days probed for the presence of the proteins in both media and cell lysates by immunoblotting against GFP (included in all clones). We found that inclusion of BDNF's SP sequence to *naked* Con-P provided the highest levels of secretion (47.4 times higher extracellular/cellular expression ratio than that of GFP), in which cells we could also visualize the clones in secretory vesicles by total internal reflection fluorescence (TIRF) microscopy (Figs. S3a–c and S10b), compared with PrePro-Con-P-SEP, which was mainly retained within cells (Fig. S10b), or GFP alone (Figs. 3a–c and S10c, green arrowhead). SP-BDNF-SEP could not be detected in culture media owing to a cross-reactive band that appeared only in the medium; but its expression was confirmed in cell extracts (Fig. S10c, orange arrowhead). Next, we infected cultured hippocampal neurons at 7 DIV via AAV1 to express SP-*naked*-Con-P-SEP under the human synapsin promoter (Hsyn). As control, we mock-infected separate neuronal cultures with AAV1-tdTomato. One week following infection, we recorded sEPSC_NMDAR_ from naïve cultures, tdTomato- or SP-*naked*-Con-P-SEP-infected neurons (Fig. 4a and b). We found that SP-*naked*-Con-P-SEP's expression significantly increased sEPSC_NMDAR_ event frequency compared with naïve and tdTomato-expressing neurons (Fig. 4c), without affecting peak amplitude or deactivation kinetics (Fig. 4d and e). We also find that expression of SP-BDNF-SEP induced a similar increase in event frequency (Fig. S10c and d). However, SP-BDNF-SEP also enhanced the average NMDAR-mediated charge transfer (i.e. larger sEPSC_NMDAR_; Fig. S10e). These observations demonstrate the strong synaptic effect of SP-*naked*-Con-P over NMDARs, comparable with the known effects of BNDF. Nevertheless, the effects instigated by SP-*naked*-Con-P-SEP diverge from the effects of the application of the purified *naked* Con-P (see Fig. 2). These likely stem from differential localizations of the two peptides, whereby the secreted form may induce a stronger effect over presynaptic activity (62). Another possibility for these differences may arise from the chronic expression (∼several days) of SP-*naked*-Con-P-SEP which may subsequently increase the number of active glutamatergic synapses (63), compared with the acute effect of the addition of *naked* Con-P to cells.

One of the main advantages of genetically encoded modulators is the ability to restrict their expression to specific cellular populations. Whereas t-toxins remain anchored to the membranes of cells to exclusively affect those cells, the synaptic release of SP-*naked*-Con-P-SEP could potentially allow the peptide to diffuse to adjacent cells. To determine whether SP-*naked*-Con-P-SEP affects nonexpressing neurons in the culture, we first infected hippocampal cultures with SP-*naked*-Con-P-SEP. Following 1 week in culture, we collected the extracellular medium (containing the secreted toxin) and applied it onto a new hippocampal culture for 48 h before recording sEPSC_NMDAR_ activity from both cultures side by side. We recapitulated the findings that neurons expressing SP-*naked*-Con-P-SEP exhibit enhanced sEPSC_NMDAR_ event frequency; however, we observed no effect in neurons treated with the enriched media (Fig. 4f and g). These results suggest that, under our experimental conditions, the concentration of the secreted *naked* Con-P that may escape the synaptic cleft is insufficient to modulate synaptic NMDARs on neurons that do not express SP-*naked*-Con-P-SEP. This outcome is to be expected, as the peptide's concentration likely decreases rapidly from the site of synaptic exocytosis due to diffusion (26, 64). Moreover, as demonstrated above, a relatively high concentration of *naked* Con-P is required to exert an effect over NMDARs. Taken together, these findings strongly suggest that *naked* Con-P is likely to have a minimal effect on NMDARs or other targets outside the synaptic cleft.

### Secreted *naked* Con-P attenuates STRO-induced apoptosis but does not protect against NMDA-induced excitotoxicity

**Fig. 5.**
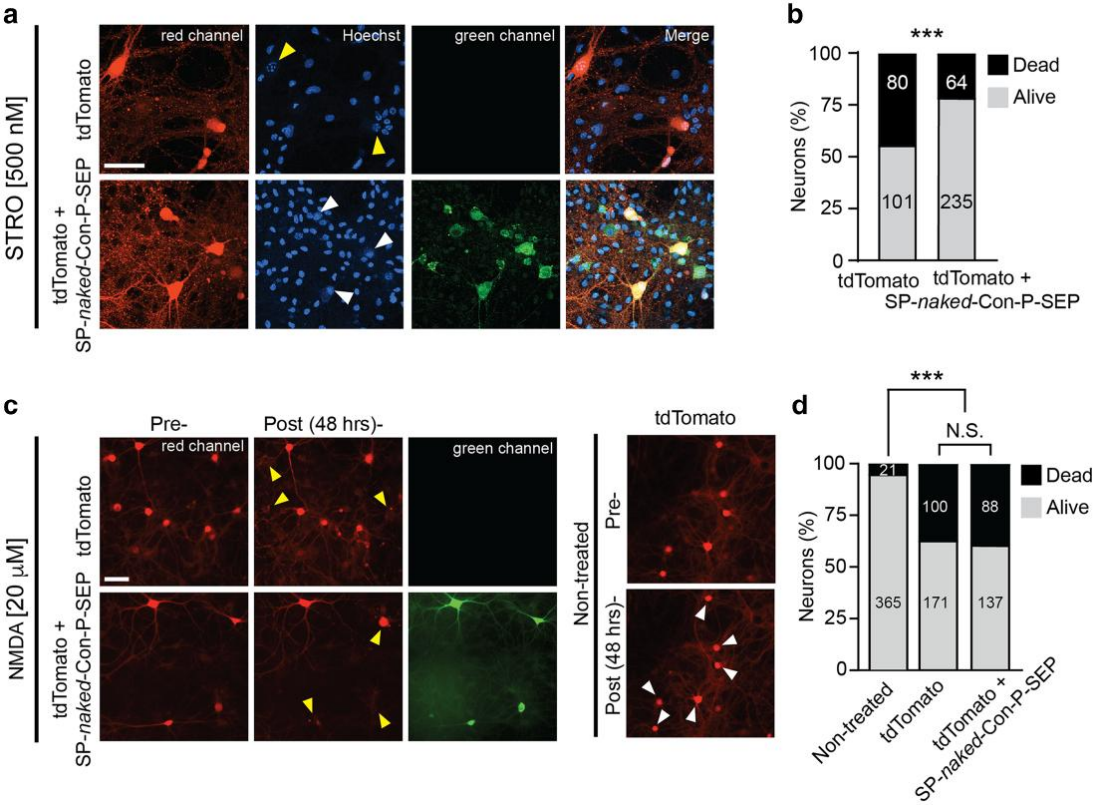
Secreted *naked* Con-P is neuroprotective against STRO-insult. a) Confocal images of mature hippocampal neurons expressing tdTomato (top row, “red channel”) or co-expressing tdTomato and SP-*naked*-Con-P-SEP (bottom row, “red channel” and “green channel”, respectively) following viral infections (Materials and methods). Neurons were exposed to STRO (500 nM). Fragmented nuclei were assessed via staining by Hoechst 33342 (“Hoechst”). Bottom and top arrows indicate healthy or apoptotic neurons, respectively. Scale bar = 20 μm. b) Summary of the fractions of STRO-induced dead neurons, in the presence or absence of SP-*naked*-Con-P-SEP. Statistical significance tested by Pearson's χ^2^ test. c) Images of live mature hippocampal neurons expressing tdTomato (top row, “red channel”) or co-expressing tdTomato and SP-*naked*-Con-P-SEP (middle row, “red channel” and “green channel”, respectively). (Right) control neurons that were not exposed to NMDA. Neurons were imaged in an incubator-based microscope (Incucyte) immediately (pre) or 48 h after (post) exposure to 20 μM NMDA for 20 min. Right and left arrows indicate locations of neurons that remained intact or underwent morphological deterioration and clearance following the insult, respectively. Scale bar = 20 μm. d) Summary of survivability (nontreated or following the NMDA insult). Number of neurons is indicated within bars. Statistical significance tested via Pearson's χ^2^ test. N.S., nonsignificant; ***P < 0.001.

We finally examined whether the expression of SP-*naked-*Con-P-SEP in neurons could reproduce the neuroprotective effect proffered by incubation of cells with the synthetic *naked* Con-P. We repeated the STRO-insult experiment over hippocampal cultures 1 week following AAV1 infection, expression, and secretion of SP-*naked*-Con-P-SEP. We co-infected neurons with tdtomato for visualizations (Fig. 5a). Expression and secretion of SP-*naked*-Con-P-SEP by the neurons themselves significantly reduced the fraction of apoptotic neurons 48 h following STRO application (Fig. 5b), supporting our observations with the purified *naked* Con-P application (see Fig. 2). We also examined the excessive NMDA-insult, in which case we did not observe any neuroprotection by SP-*naked*-Con-P-SEP (Fig. 5c and d). Thus, SP-*naked-Con-P* promotes neuronal survival specifically against deterioration inflicted by loss of synaptic NMDAR activity and not against excessive glutamate-driven excitotoxicity.

## Discussion

Here, we report a novel synthetic *naked* Con-P peptide (lacking Gla residues) that embodies unique pharmacological properties toward GluN2A- and GluN2B-containing receptors. This discovery led us to the engineering of a genetically encoded secreted *naked* variant (denoted SP-*naked*-Con-P) that is readily secreted by primary hippocampal neurons, for cell-autonomous delivery of the peptide to neuronal synapses. There, secretion of SP-*naked*-Con-P induces highly specific modulations of endogenous synaptic NMDARs and proffers neuroprotection against STRO insults. This strategy has yet to be demonstrated for NMDARs.

The development of SP-*naked*-Con-P was preceded by our scrutiny of the functionality of *naked* Con-G variants, in which case we immediately noted that the *naked* peptides lose their selectivity and potency toward GluN2B-containing receptors (Fig. 1). We remained motivated to explore *naked* Con-P owing to its unique sequence that includes cysteines unlike all other Cons. Indeed, *naked* Con-P remained inhibitory of GluN2Bs, although to a lesser extent and in a noncompetitive manner, analogous to *naked* Con-G. Unexpectedly, however, it garnered the ability to potentiate GluN2A-containing receptors (unlike *naked* Con-G and many other variants examined; Figs. 1a–d, S1b, and S2c). This potentiation is likely induced by a concerted structural change in more than one domain within or between the GluN2A subunit (65). This is supported by the inhibition of *naked* Con-P over triheteromeric GluN1a/GluN2A/GluN2B receptors (Fig. S5c–e). We also find that the potentiating effect of *naked* Con-P is highly dependent on the presence of glutamate (Fig. 1e and f), whereby *naked* Con-P potentiates currents induced by subsaturating—but not saturating—glutamate concentrations (Figs. 1f and S1a). These are highly reminiscent of the actions of PAMs (28, 29, 66, 67). Together, these observations are compatible with known mechanisms of many NMDAR-modulating molecules that bind at locations found specifically in the ATD that is the modulatory domain of the receptor (1, 29). This domain also shows greater sequence variability between the GluN2 subunits, whereas the LBD is highly conserved. This helps to explain the divergence between the activity of *naked* Con-P over the four different GluN2A-D subunits (Figs. 1b, d and S5a, b). Interestingly, the con-ikot-ikot toxin is suggested to bind at the interface between the ATD and LBD of the AMPAR subunit (a close relative to NMDARs) and is suggested to promote opening of the receptor (68, 69). However, it must be noted that the latter toxin is much longer than Cons and can therefore bridge between the very large distances between the domains (68, 69). This is unlikely to be achieved by Cons, but it certainly raises the possibility that Cons can enter this region in the NMDAR (highly water soluble) to affect receptor motion and, thus, gating.

The above attributes motivated us to explore *naked* Con-P's effect over neuronal function and protection against NMDAR-relevant insults. *Naked* Con-P proved to be efficacious in inhibiting NMDAR-dependent currents in young neurons (Fig. 2c). Although the extent of this inhibition (∼20%) is on par with the inhibition of GluN2B-containing receptors (Fig. 1d), it may also arise from the very mild inhibition of GluN2D-containing receptors that may be present in these young neurons (Fig. S5b). Conversely, *naked* Con-P potentiated sEPSC_NMDAR_ in mature neurons (Fig. 2c–e). This potentiation is moderate compared with the extents observed in HEK293T cells or in oocytes expressing GluN2A diheteromers, and this may be the result of the many other NMDAR subtypes present in mature neurons, notably triheteromeric NMDARs. Nevertheless, the moderate potentiation of synaptic activity by *naked* Con-P was sufficient to rescue neurons from degeneration following a STRO insult (Fig. S8).

The above demonstrations led us to explore two genetic strategies for delivering *naked* Con-P to neurons. We initially explored the tethered-toxin approach (as in Ref. (12)); however, these attempts failed to yield any observable modulation of NMDARs (Fig. S9). This led us to pursue a different cellular route for toxin expression and delivery to NMDARs at the membrane, and specifically at the synapse. We focused on secretion of the peptides, as neurons are prototypical secreting cells and rely on secretion of many different peptides, for instance neurotrophins (70). One such peptide is BDNF, known to be secreted directly into the synaptic cleft (60). We thereby deemed this secretory route ideal for targeting *naked* Con-P to synaptic receptors, where GluN2A-containing receptors predominate. We introduced the SP from BDNF and found it to be sufficient for enabling strong secretion of the peptide by differentiated PC-12 cells (Figs. 3 and S10a, b; SP-*naked*-Con-P). We go on to show that viral expression of SP-*naked*-Con-P-SEP in hippocampal neurons results in promoting an increase in the frequency of NMDAR-mediated synaptic events (Figs. 4 and S10c–e). Notably, the acute application of *naked* Con-P and the chronic expression of SP-*naked*-Con-P-SEP in cultured neurons resulted in facilitation of different aspects of synaptic NMDAR events. Explicitly, *naked* Con-P increased peak amplitude, and shortened deactivation kinetics, of NMDAR-mediated sEPSC_NMDAR_ (Fig. 2e and l), pointing toward a direct modulation of postsynaptic activity of GluN2A-containing receptors. Inversely, its prolonged (∼week) expression and secretion did not show a direct effect on sEPSC_NMDAR_ amplitudes or deactivation kinetics, rather significantly increased their frequency, strongly implying a presynaptic effect. Nevertheless, we cannot overrule that these divergences may also be attributed to additional factors. For instance, it is challenging to compare the effects of these two different administration routes as the concentrations of the secreted peptides are unknown, which is likely to exhibit variations in activation (and inhibition) of receptors populations within or outside the synaptic cleft (i.e. postsynaptic, presynaptic, and extrasynaptic). Furthermore, there is a very large difference in the exposure time of the receptors to the different peptides: minutes exposure to purified peptides compared with days/weeks to the secreted variants. This alone could trigger compensatory mechanisms. Regardless, in both instances, we observe a strong neuroprotective effect by *naked* Con-P. The collective features of Cons, namely being the only known NMDAR-selective peptide toxins, their small size (71), and amenability to genetic encoding (as shown here) highlight their vast potential for genetic engineering (Figs. 3–5). In fact, it is suggested that hundreds of thousands of active peptides remain undiscovered in the venoms of different Conus snails. These could potentially harbor additional NMDARs peptide modulators. Here, we provide key attributes that should be useful when screenings for such compounds, for instance cysteines residues and reduced Gla-content. We also envision that machine learning–based protein structure prediction should accelerate the repertoire of viable, subunit-selective, and more potent variants; highly suitable for gene-delivered therapeutics for brain pathologies involving NMDAR-dependent imbalances (72–74). We envision that our proposed method could be applied to animal models in which the activity and expression of GluN2A-containing receptors declines in various conditions, such as schizophrenia and aging (29, 75–77). In these instances, the prolonged effect of a diffusive agent, such as the secreted *naked* Con-P, may be ideal for continuously negating these declines. In fact, this approach is behind the motivation to pursue GluN2A-selective PAMs to treat the abovementioned disease or age-related declines in GluN2A's (expression and/or activity) (29, 78), and other diseases such as Dravet syndrome and AD (79).

## Materials and methods

### Ethical approval

Animal experiments were approved by the Technion Institutional Animal Care and Use Committee (permit numbers: IL-129-09-17, IL-162-10-21, IL-130-09-2017, and IL-161-10-21), and all experiments strictly followed the approved guidelines.

### Peptides and reagents

All Conantokins used in this study were provided by Alomone Labs, according to peptide sequences designed by us, following folding and HPLC purification (see Table 1 for peptide sequences). Lyophilized peptide powders were kept desiccated and protected from light in room temperature until dissolved in recording solutions immediately prior to experimentations. All receptor blockers, modulators, and NGF (cat# N-240) were also provided by Alomone Labs and were dissolved and stored according to the manufacturer's instructions.

### cDNA cloning, mRNA, and AAV production

cDNA plasmids were either gifts from other groups or purchased from Addgene and processed in-house using PCR and DNA purification methods. mRNA and viral particles were made in-house using commercially available tools (see Supplementary Materials and methods).

### Electrophysiological recordings in *Xenopus oocytes* and cell lines

Whole-cell channel activity was measured from oocytes and mammalian cells (HEK293T, hippocampal neurons) by either two electrode voltage clamp (TEVC) or patch-clamp techniques following heterologous channel expression (for elaborated cell culture maintenance, expression methods, and recording criteria, see Supplementary Materials and methods).

### Confocal macroscopy and live-cell imaging

Cell culture imaging of fixed/stained cells as well as live cells expressing calcium indicators was done using a confocal laser scanning microscope (LSM-900, ZEISS). NMDA excitotoxicity was imaged by an IncuCyte ZOOM live-cell analysis incubator (see Supplementary Materials and methods).

### Data analysis and statistics

Electrophysiological data was analyzed using Clampfit (Molecular Devices). Confocal images were acquired using ZEN black (ZEISS) and analyzed using Fiji (ImageJ). Statistical tests were conducted using Prism 8 (GraphPad). For elaboration on analyses and tests performed in this study, see Supplementary Materials and methods.

## Supplementary Material

pgaf041_Supplementary_Data

## Data Availability

All data are included in the manuscript and/or Supplementary material.
