# Supplementary material for: A genetically encoded secreted toxin potentiates synaptic NMDA receptors in hippocampal neurons and confers neuroprotection
Source: PNAS Nexus. 2025 Feb 6;4(2):pgaf041. doi: 10.1093/pnasnexus/pgaf041 (PMC11826341; doi:10.1093/pnasnexus/pgaf041)
Supplement: pgaf041_Supplementary_Data [file pgaf041_supplementary_data.docx]

**Supplementary** **Materials and Methods**

*cDNA cloning, mRNA and AAV production*

cDNA plasmids encoding genes of NMDAR subunits (GRIN1, GRIN2A, GRIN2B, GRIN2A (N-2B), GRIN2B (N-2A)), t-Cons (NoTox, t-Con-G, t-Con-P), and GFP were used to tag and express functional receptors and membrane-tethered toxins in *Xenopus* oocytes and HEK293T cells. In vitro transcription of mRNA for the expression of receptors in oocytes was produced from pGEM-HE/J plasmids, as previously described^33,95^. Briefly, cDNAs were linearized, and mRNA was transcribed using mMESSAGE mMACHINE transcription kit (Invitrogen™), aliquoted and stored at -80°C. The rat pGEM GluN1a and GluN2A-D clones for expression in oocytes were obtained from Ehud Y. Isacoff^34^, the pci-neo human GluN1a and GluN2B clones for expression in HEK293T cells from Tal Garin-Shkolnik^33^, rat pGEM GluA1/2 clones from Yael Stern-Bach, and the mouse pGEM GIRK1/2,GABAb1 and mGluR2 clones from Nathan Dascal. The rat pGEM clones for 1/2A*/2B* triheteromeric NMDARs were a gift from Kasper Hansen^50^. The rat 2A-(N-2B) and GluN2B-(N-2A) clones were a gift from Pierre Paoletti^53^. The BDNF-SEP clone was a gift from James O. McNamara (Addgene, plasmid #83955). Subcloning of clones into a pAAV backbone was performed by enzymatic restriction between HindIII and XhoI. pAAV-CAG-tdTomato was purchased from Addgene (plasmid #59462). For SP-*naked*-Con-P-SEP containing plasmids, the BDNF sequence was replaced with that of Con-P by reverse PCR using the following primers:

For SP-naked-Con-P-SEP: 5-AGGCACTCCTGGTATTTGCTGTGCTCTTCTTCGCCcgccttcatgcaaccaaagtat-3;

5-GAGGGAGATCAGAGTCAACAAGGTGCAGCAAGAATGTggatccgactacaaggatgac-3;

For PrePro-Con-P-SEP:

5-TCAGGCACTCCTGGTATTTGCTGTGCTCTTCTTCGCCgcgccggaccctcatgga-3;

5-GGGAGATCAGAGTCAACAAGGTGCAGCAAGAATGTggatccgactacaaggatgac-3;

Plasmids for the expression of membrane-tethered toxins were based on a membrane-expressing MInDI clone ( Addgene Plasmid #41732), which was modified by us to include tdTomato and ER exit motifs as reported by us^17^. t-Con-G and t-Con-P were introduced within the clone by reverse PCR using the following primers:

For t-Con-G: 5-ATTCTCTTGGAGCTCCTCCTCACCagatctGTCACCAGTGGAACCTGGAA-3;

5-CAAGAGCTCATTCGCGAGAAATCCAATCTGCAGGTCGACGAACAAAAACT-3;

For t-Con-P:

5-CAGGCACTCCTGGTATTTGCTGTGCTCTTCTTCGCCAGATCTGTCACCAGTGGAACCTGG-3;

5-AGGGAGATCAGAGTCAACAAGGTGCAGCAAGAATGTCTGCAGGTCGACGAACAAAAACTC-3.

All PCR products were verified by Sanger sequencing. The final protein sequences of genetically-encoded naked Con-P are as follows:

t-Con-P: METDTLLLWVLLLWVPGSTGDRSGEEEHSKYQECLREIRVNKVQQECLQVDEQKLISEEDLNASGGGGSGGGGSGGGGSTGAVGQDTQEVIVVPHSLPFKVVVISAILALVVLTIISLIILIMLWQKKPRSAAARGSSSGSSSMVSKGEEVIKEFMRFKVRMEGSMNGHEFEIEGEGEGRPYEGTQTAKLKVTKGGPLPFAWDILSPQFMYGSKAYVKHPADIPDYKKLSFPEGFKWERVMNFEDGGLVTVTQDSSLQDGTLIYKVKMRGTNFPPDGPVMRKKTMGWEASTERLYPRDGVLKGEIHQALKLKDGGHYLVEFKTIYMAKKPVQLPGYYYVDTKLDITSHNEDYTIVEQYERSEGRHHLFLYGMDELYKKSRITSEGEYIPLDQIDINVGGSGFCYENEVRGRSWTY*

SP-naked-Con-P-SEP:

MTILFLTMVISYFGCMKAGEEEHSKYQECLREIRVNKVQQECGSDYKDDDDKSKGEELFTGVVPILVELDGDVNGHKFSVSGEGEGDATYGKLTLKFICTTGKLPVPWPTLVTTLTYGVQCFSRYPDHMKRHDFFKSAMPEGYVQERTIFFKDDGNYKTRAEVKFEGDTLVNRIELKGIDFKEDGNILGHKLEYNYNDHQVYIMADKQKNGIKANFKIRHNIEDGGVQLADHYQQNTPIGDGPVLLPDNHYLFTTSTLSKDPNEKRDHMVLLEFVTAAGITHGMDELYK*

PrePro-Con-P-SEP:

MTILFLTMVISYFGCMKAAAGGYPYDVPDYAGAPMKEANIRGQGGLAYPGVRTHGTLESVNGPKAGSRGLTSLADTFEHVIEELLDEDQKVRPNEENNKDADLYTSRVILSSQVPLEPPLLFLLEEYKNYLDAANMSMRVRRGEEEHSKYQECLREIRVNKVQQECGSDYKDDDDKSKGEELFTGVVPILVELDGDVNGHKFSVSGEGEGDATYGKLTLKFICTTGKLPVPWPTLVTTLTYGVQCFSRYPDHMKRHDFFKSAMPEGYVQERTIFFKDDGNYKTRAEVKFEGDTLVNRIELKGIDFKEDGNILGHKLEYNYNDHQVYIMADKQKNGIKANFKIRHNIEDGGVQLADHYQQNTPIGDGPVLLPDNHYLFTTSTLSKDPNEKRDHMVLLEFVTAAGITHGMDELYK*

AAV viruses were produced by the iodixanol method, as previously described^96^. Briefly, HEK293T cells (human embryonic kidney cells, ATCC #CRL-1573) were grown in Dulbecco’s modified Eagle’s medium/nutrient mixture F-12 (supplemented with 10% fetal bovine serum and 1% L-glutamate) on 10 ml tissue culture plates (Corning, Cat. 430167) at 37 °C and 5% CO_2_. Cells were grown to 70%-80% confluency and transfected with three viral plasmids (Helper, rep/cap, and transfer) using polyethyleneimine (PEI) at a ratio of 8.1, 5.4, and 13.5 µg of DNA, respectively. After 6–8 h, the growth media was replaced by serum-free media supplemented with 1% Glutamax. Media were collected after 48 and 72 h following transfection (collected media were preserved at −80 °C). The collected media was then filtered and concentrated via the iodixanol step gradient method to a final volume of 100–500 μl. The viral titer was determined by qPCR. Only viruses with a titer of >10^12^ were used.

*Western blotting*

Proteins from differentiated PC-12 cells were extracted by 1% N-dodecyl-maltoside (Sigma) with 0.1 mg/mL PMSF and protease inhibitor cocktail (Tivan Biotech) (1:100) in PBS for 1 hour on ice, followed by centrifugation at 4°C and 16,000 g for 30 minutes. Cell extracts and filtered medium samples were mixed with loading dye, denatured at 100°C for 10 minutes, and separated by 10% SDS-PAGE followed by transfer onto a nitrocellulose membrane (33 mAmp overnight at 4°C). Membranes were washed with TBSx1 and blocked for 1 hour with 5% nonfat dry milk in TBS-T [20 mM Tris, 137 mM NaCl (pH 7.6), supplemented with 0.1% Tween 20], washed with TBS-T and probed with primary antibody overnight at 4°C (anti–GFP (1:1000); Cell Signaling Technology). After 3 washes with TBS-T, membranes were incubated with secondary antibody (anti-mouse HRP; Jackson ImmunoResearch) for 1 hour at R.T. Membranes were washed again, and immunostaining was visualized by ECL (enhanced chemiluminescence). Densitometry of bands was performed by Fiji (ImageJ). Gel films were scanned, and band intensities were measured after background subtraction. The presented quantifications are the average ratio of medium-to-cell band intensities for each independent experiment per group.

*Xenopus oocytes*

Adult *Xenopus* frogs were anesthetized using 0.5% Ms-222, and a minor abdomen cut was made to extract a portion of the ovary. Oocytes were folliculated by treatment with collagenase and then kept in NDE-96 solution^97^ (in mM: 96 NaCl, 2 KCl, 1 MgCl_2_, 1.8 CaCl_2_, 5 HEPES. pH = 7.4. 100 µg/mL penicillin, 100 µg/mL streptomycin). One day after surgery, oocytes were injected with 50 nL mRNA each (1:1 GluN1:GluN2A/B) and incubated at 18°C for 1-3 days before recordings.

*Cell culture*

HEK293T cells (human embryonic kidney cells, ATCC #CRL-1573) were grown on Corning 100-mm dishes in Dulbecco’s modified Eagle’s medium (DMEM, Biological Industries™) in a humidified incubator at 37°C and 5% CO2. Prior to transfection, cells were moved to 35-mm plates for overnight incubation and then transfected for expression using ViaFect transfection reagent (Promega™) and 1-2 µg cDNA per plate. Six hours after transfection, the cells were moved to 24-well plates containing PDL-covered glass coverslips, and 200 µM AP5 and 2 µM MK-801 were added to prevent NMDAR-induced toxicity. The cells were then incubated 1-2 days prior to recordings.

Hippocampal neurons were extracted, as described previously^98^. Briefly, eight hippocampi of four-six neonate (day 0/1) rats (from various females) were extracted, and 100,000 cells per well were plated in 24-well plates containing PDL-covered glass coverslips and MEM (Gibco™)-based neuronal growth medium (NGM, see below). Cultures were kept in a humidified incubator at 37°C and 5% CO2. On DIV 5 and twice a week thereafter, the culture medium was supplemented with ARA-C [4 µM] to suppress glial proliferation.

*Electrophysiological recordings*

Oocytes were recorded using a two-electrode oocyte clamp OC-725C amplifier (Warner Instruments) and Digidata 1550B digitizer (Axon Instruments). Signals were digitized at 1000 Hz. For dose-response and competitiveness experiments, oocytes were gravity-perfused in room temperature (25°C) (RT) BARTH solution (in mM): 100 NaCl, 0.3 BaCl_2_, 5 HEPES, (pH adjusted with 2.5 mM KOH to 7.3)^34,49^ and voltage-clamped at -60 mV.

HEK293T cells and hippocampal neurons were whole-cell voltage clamped at -70 mV using a Multiclamp 700B amplifier and Digidata 1440A digitizer patch system (Axon Instruments) with 5-10 MΩ glass pipettes filled with intracellular solution (in mM: 135 K-gluconate, 10 NaCl, 10 HEPES, 2 MgCl2, 2 Mg-ATP, 1 EGTA, pH = 7.3). For GABAaR-mediated sIPSCs, intracellular solution was changed to a high-chloride solution (in mM: 135 CsCl, 10 HEPES, 1 EGTA, 1 Na-GTP, 4 Mg-ATP and 2 QX-314 (pH 7.4, adjusted with KOH). Evoked currents from HEK293T cells and sEPSCs/sIPSCs from neurons were digitized at 1000 and 20000 Hz, respectively, as previously reported^17^. Cells were gravity perfused in RT with a physiological recording solution (in mM: 138 NaCl, 1.5 KCl, 2.5 CaCl_2_, 10 D-glucose, 5 HEPES, 0.05 glycine, pH = 7.4). For recording NMDAR-mediated EPSCS, Mg^2+^ was omitted from the extracellular solution, whereas for recording GABAaR-mediated sIPSCs, the extracellular solution contained (in mM): 140 NaCl, 5 KCl, 0.8 MgCl_2_ or 2 MgCl_2_, 10 HEPES, 10 glucose (pH 7.4, adjusted with NaOH). Series resistance and neuronal resting membrane potential (rVm) were monitored and documented at the start and end of recordings. Series resistance was 10-35 mΩ and neuronal rVm was -80 - (-55) mV. Cells that did not meet these criteria or that deviated more that 20% from initial values were omitted from further analysis. During steady-state recordings, baseline and epochs of wash-in solutions were established by current equilibrium for a minimum of 15 seconds following each change in wash solution (solutions were continuously perfused). For sEPSC recordings, baseline and treatment epochs were recorded for 3-5 minutes each with continuous perfusion.

*Hippocampal neuron and HEK293T cell fixation, permeabilization and staining*

For neuronal cultures, 48 hours following *STRO* application, the medium was replaced with 4% paraformaldehyde (PFA) in phosphate-buffered saline (PBS) for 15 minutes in R.T., followed by three washes with PBS. Cells were then permeabilized with Triton-X (1:1000, 15 minutes at RT) and washed with PBS. PBS was replaced by Hoechst 33342 (10 mg/ml) diluted in PBS (1:2000) for 10 minutes in R.T. and protected from light, followed by three washes with PBS. Finally, the cells were stained using Neurotrace™ 640/660 far-red, fluorescent Nissl stain (Invitrogen™). Cell plates were stored at 4°C and protected from light until use.

HEK293T cells expressing t-Cons were fixated similarly to neuronal cultures, omitting permeabilization, 48 hours following transfection. Cells were stained against the Myc epitope to visualize the presence of t-Cons on the plasma membrane.

*Confocal fluorescence imaging*

Imaging was performed using a confocal laser scanning microscope (LSM-900, ZEISS). Coverslips were mounted on the microscope chamber in a corning 35-mm plastic cap filled with physiological recording solution (for Ca^2+^ imaging experiments) or PBS (for fixated HEK293T cells and neuronal death assay experiments) and imaged with a W-Plan APOCHROMAT 20x/1.0 DIC (UV) VIS-IR M27 75 mm objective. Hoechst 33342-stained nuclei fluorescence was excited by a 375 nm laser, anti-Myc secondary antibody was excited by a 488 nm laser, and Nissl-stained body fluorescence was excited by a 640 nm laser. Neuronal death assay images were obtained by Z-stack recordings to capture somata and nuclei fluorescence from each neuron. For Ca^2+^ imaging experiments, GCaMP7f fluorescence was excited by a 488 nm laser.

*Live-cell imaging*

Dendritic calcium fluorescence of GCamP7f-expressing hippocampal cultures was continuously imaged by an LSM-900 488 nm laser in 5-minute intervals for baseline, toxin application and wash phases. Active dendrites were manually and randomly selected by detectable fluorescence in the baseline phase of recordings.

Hippocampal cultures tested for NMDA excitotoxicity were imaged by an IncuCyte® ZOOM live-cell analysis incubator using a X10 objective in both red and green channels for 48 hours. Each well was imaged every 4 hours for 25 regions of interest. The cell count was determined manually by comparing the start time and 48-hour images.

*Data analysis and statistics*

All electrophysiological data were analyzed by Clampfit (Molecular Devices). Con-G IC_50_ value was calculated using SigmaPlot (Systat Software inc.) by fitting the data to an adapted Hill equation: Response = 1 – [Con-G]^nH^ (IC_50_^nH^ + [Con-G]^nH^). [Con-G] indicates Con-G concentration (μM), and nH is the Hill slope. IC_50_ was defined as the Con-G concertation required to induce half of the maximal response. In all TEVC recordings in oocytes, the responses were normalized to current amplitude obtained from that individual cell. Neuronal death assay confocal imaging data were acquired by ZEN black (ZEISS) and analyzed by the cell counter plugin of Fiji (ImageJ). The IncuCyte® image cell count was determined manually using Fiji. For dendritic calcium analysis, a customized MATLAB (MathWorks®) script was used to detect calcium transients and compute signal size as DF/F. All statistical tests were conducted using Prism 8 (GraphPad). All data except neuronal death ratios are presented as the mean ± SEM. The number of cells in each group is presented as n (oocytes, neurons), and the number of independent experiments is presented as N. For oocytes, N represents the number of independent batches extracted from different frogs. For HEK293T and PC-12 cells, N represents number of independent batches used for transfection and independent experiments. For cultured hippocampal neurons, N represents the number of independent cultures used, where each culture was obtained by extraction of cells from hippocampi of at least four neonate rats. Statistical significance was obtained by *Student’s T*-test (for two-group comparisons) or one-way or two-way ANOVA for multiple group comparisons with Tukey’s post hoc test. Statistical significance for the neuronal death ratio was obtained by the Pearson’s chi-squared test with Yate’s correction. For reports involving two or more independent experiments (N > 2), pooled data of all cells (n) was tested for normal distribution of the population, before further analysis. **p* < 0.05; ***p* < 0.01, and ****p* < 0.001; N.S., non-significant.

**Supplementary Figures**


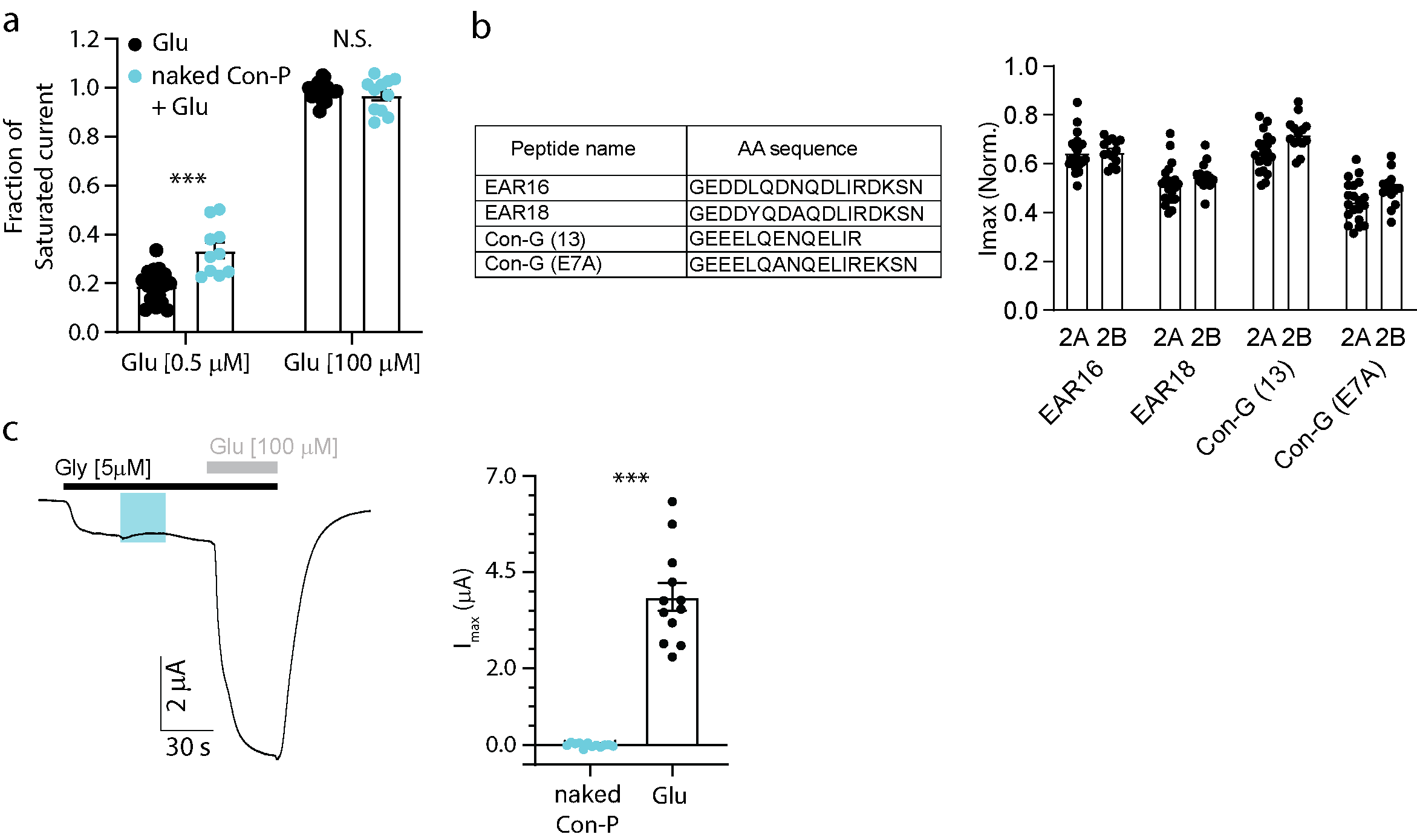


**Supplementary Figure 1. *Naked* Con-P, but not *naked* Con-G, potentiates GluN2A-containing receptors by a glutamate concentration-dependent mechanism. a.** Fraction of current evoked in the presence or absence of 50 μM *naked* Con-P by 0.5 μM or 50 μM glycine and glutamate in comparison to the maximal current evoked by saturating agonist concentration (100 μM). Statistical significance tested by two-way ANOVA, followed by *post hoc* Tukey test. **b.** Multiple *naked* Con-G variants inhibit GluN2A and -2B-containing receptors. (left) Sequences of full length (15 amino acids) or truncated (13 aa) *naked* Con-G variants, with aspartate or alanine substitutions (EAR16, EAR18 and E7A, respectively). (right) Summary of the effect of the peptides (at 100 μM) over GluN2A- (2A) or GluN2B-containing (2B) receptors expressed in oocytes. Receptors were activated by glycine and glutamate (5 μM). **c.** *Naked* Con-P is not a partial agonist. A representative trace from *Xenopus* oocyte expressing GluN2A-containing receptors, activated by 5 μM glycine (black bar). Applications of *naked* Con-P (50 μM, cyan bar) and glutamate (100 μM, grey bar) are indicated; summarized on the right. Statistical *s*ignificance was tested by paired T-test. N.S., non-significant; *** p< 0.001.


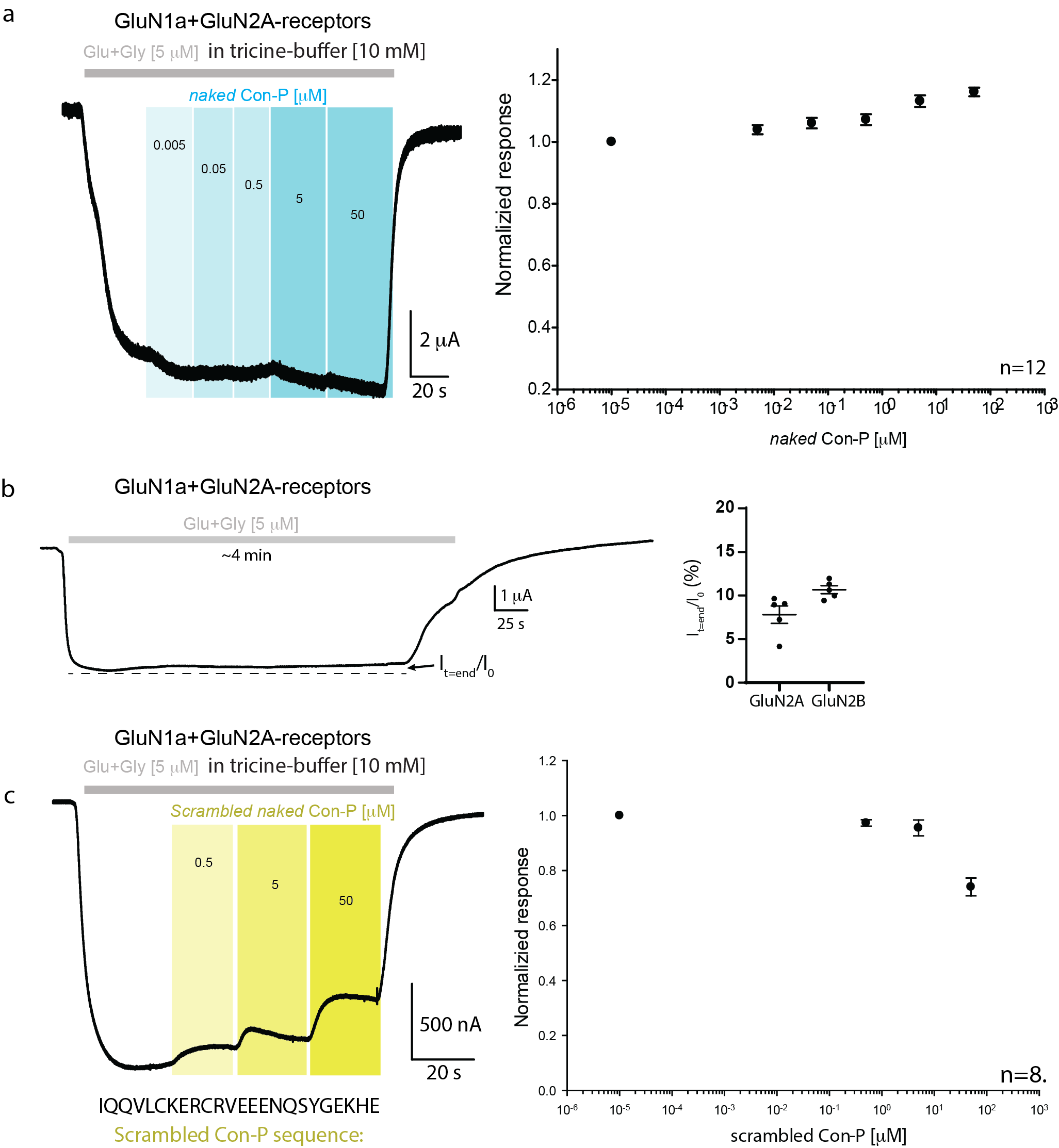


**Supplementary Figure 2. Potentiation of GluN2A-containing receptors is not artefactual. a.** Representative traces from *Xenopus* oocytes expressing GluN2A-containing receptors following continuous application of 5 μM glycine and glutamate (grey bars) in tricine buffer (10 mM), and incrementing concentrations of *naked* Con-P (cyan gradient); summarized on the right. **b.** Representative trace from *Xenopus* oocytes expressing GluN2A-containing receptors showing the decline in current following continuous application of 5 μM glycine and glutamate (grey bar) for >4 minutes; summarized on the right. **c.** Representative trace from *Xenopus* oocytes expressing GluN2A-containing receptors following continuous application of 5 μM glycine and glutamate (grey bar), and incrementing concentrations of scrambled *naked* Con-P (yellow gradient) recorded in tricine-buffer (10mM).


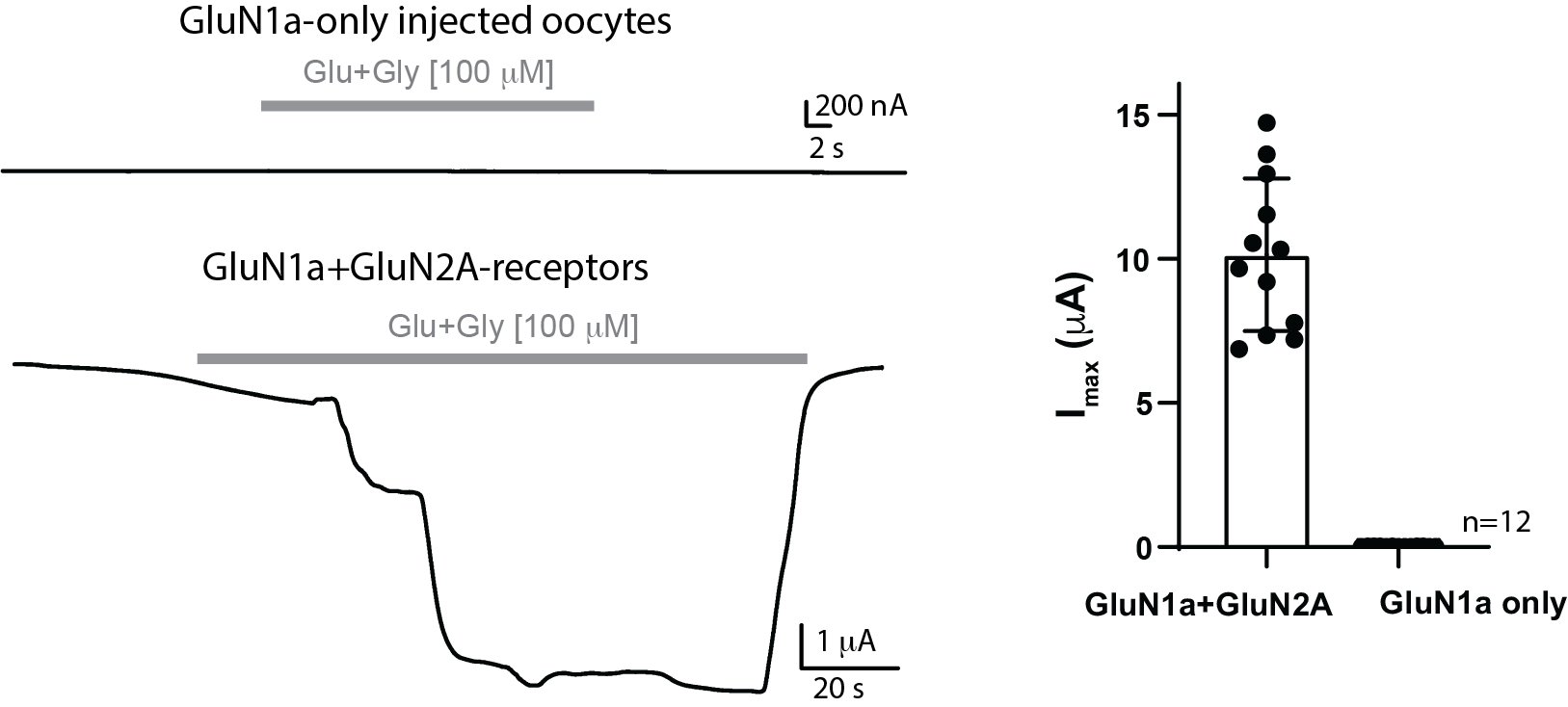


**Supplementary Figure 3. Potentiation of GluN2A-containing receptors is not contributed by non-canonical NMDARs.** Representative traces from *Xenopus* oocytes expressing mRNA of GluN1a alone (top) or GluN1A and GluN2A (bottom). Receptors were activated by continuous application of glutamate and glycine; summarized on the right.


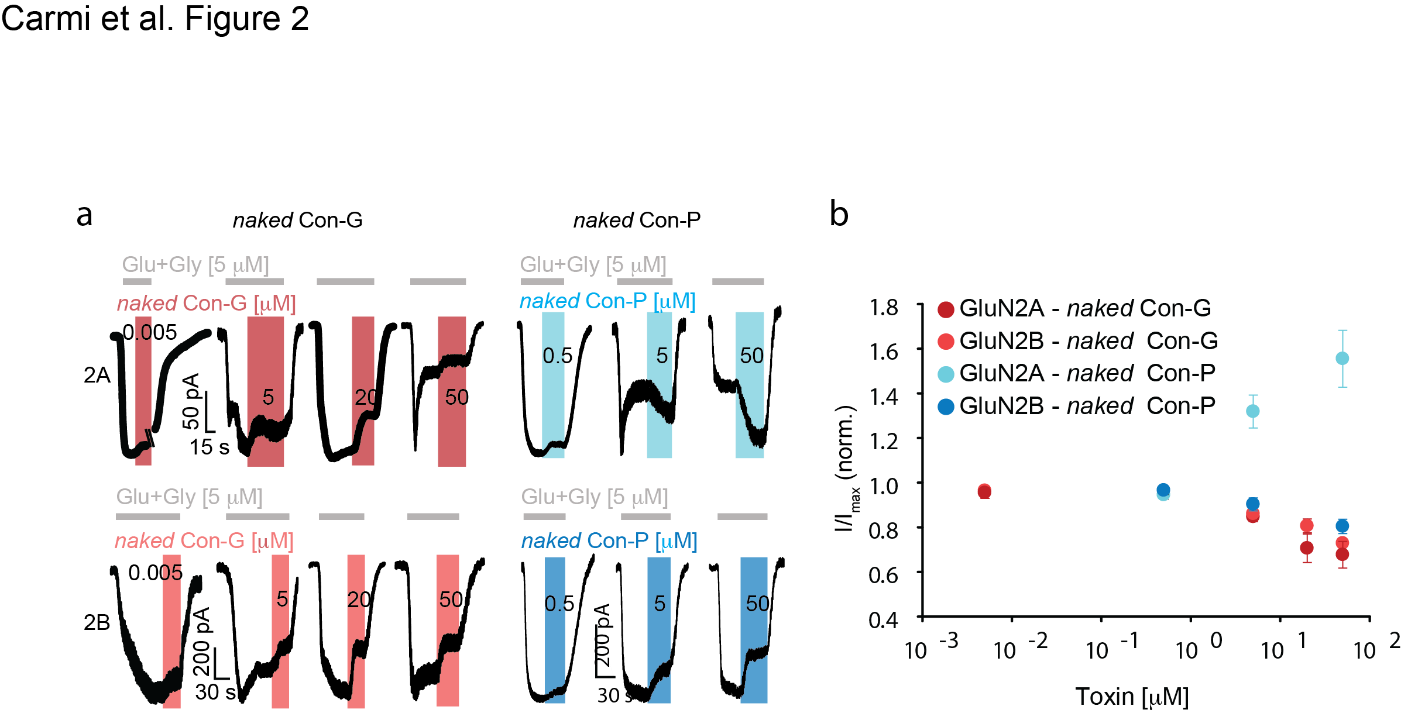


**Supplementary figure 4. Pharmacological characterization of the effect of *naked* Conantokins on NMDARs in HEK293T cells. a.** Representative traces from HEK293T cells expressing GluN2A- (top traces) or GluN2B-containing receptors (bottom), following application of 5 μM glycine and glutamate (grey bars), and incrementing concentrations of *naked* Con-G (left; crimson and red bars) or *naked* Con-P (right; cyan and blue bars). Each trace shows the effect of one toxin concentration in separate cells (0.005 μM, 0.5 μM, 5 μM, 20 μM, 50 μM), to avoid desensitization of the current by repeated ligand applications. **b.** Dose-response curves for toxins screened in HEK293T cells.


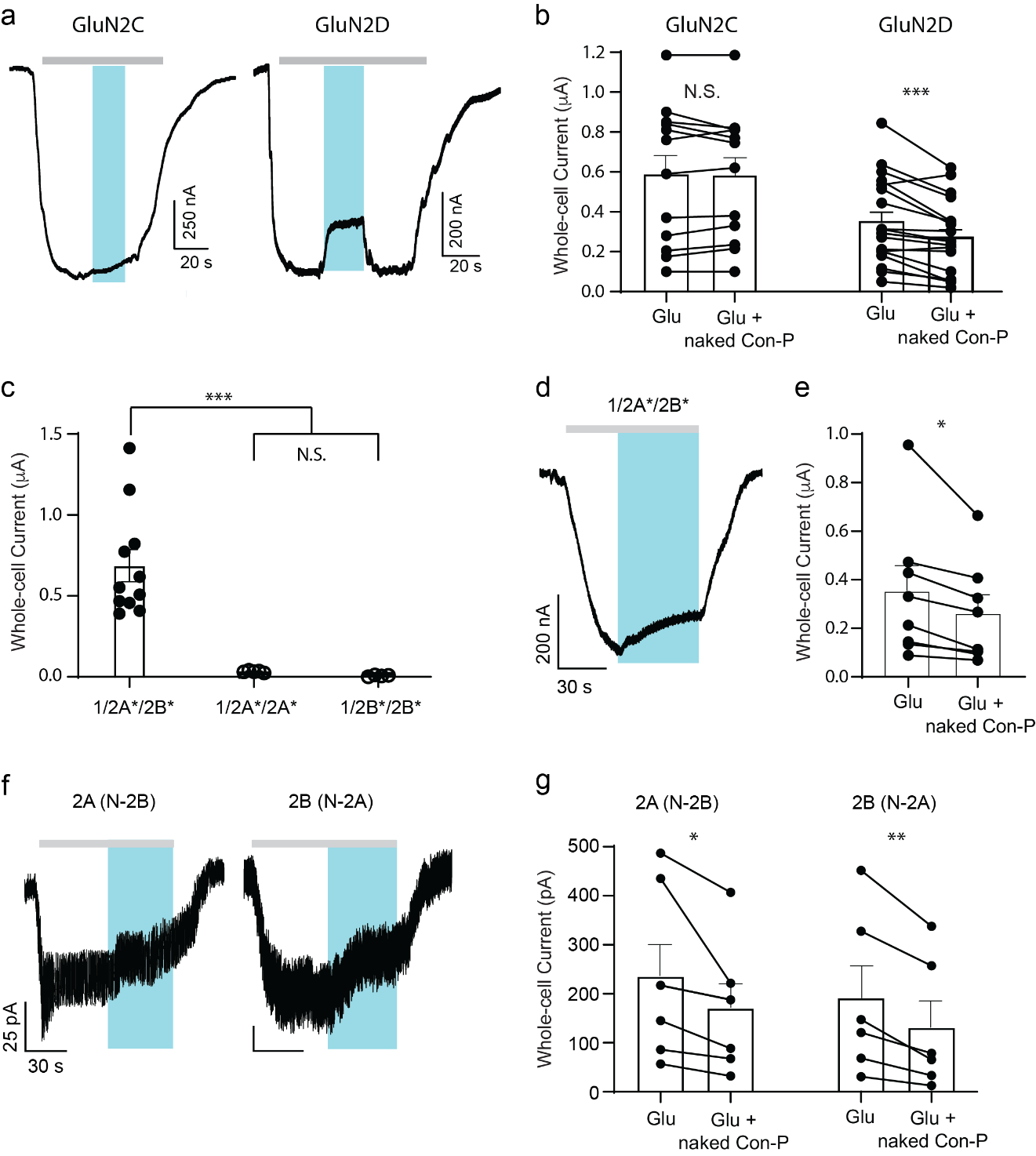


**Supplementary Figure 5. *Naked* Con-P specificity towards NMDAR subtypes. a.** Representative traces from *Xenopus* oocytes expressing GluN2C- or GluN2D-containing receptors, following application of 5 μM glycine and glutamate (grey bars), and *naked* Con-P (50 μM, cyan bar); summarized in (**b**). *Naked* Con-P partially inhibits GluN2D-containing receptors, with no significant effect over GluN2C-containing receptors. Statistical significance tested by paired T-tests. **c-e.** *Naked* Con-P exerts intermediate inhibition over GluN2A and -2B tri-heteromers. Receptor activation (by 100 μM glutamate and glycine) is only obtained when both the GluN2A* and GluN2B*-clones are co-injected into oocytes (**c**) (2A*/2A* or 2B*/2B* di-heteromers do not reach PM due to ER retention motifs, denoted by asterisks, and see ^51^). Statistical significance tested by One-way ANOVA followed by *post hoc* Tukey test. **d**. Representative trace showing 2A*/2B* tri-heteromeric currents elicited by obtained by 5 μM glycine and glutamate (grey bar), and the inhibitory effect of *naked* Con-P (50 μM, cyan bar) ; summarized in (**e**). Statistical significance tested by paired T-test. **f, g.** Effect of *naked* Con-P over chimeric NMDARs. Representative traces (**f**) and summary (**g**) of the effect of 50 μM *naked* Con-P (cyan) over the currents from chimeric NMDARs expressed in HEK293T cells, in which the amino terminal domains have been swapped between the GluN2A and -2B subunits. GluN2A-subunits with the amino terminal domain from GluN2B are denoted as GluN2A (N-2B) and GluN2B (N-2A) are GluN2B-containing receptors with the amino terminal domains from GluN2A. Note that the replacement of the amino terminal domain of GluN2A by that of GluN2B renders *naked* Con-P weakly, albeit significantly, inhibitory of the 2A receptor instead of potentiating it. Likewise, the inhibitory effect of *naked* Con-P is significantly dampened in GluN2B (N-2A)-containing receptors (see **Fig. 1 and Table 2**). Receptors were activated by 5 μM glycine and glutamate (grey bar). Statistical significance of repeated measures was tested by Two-way ANOVA followed by *post hoc* Tukey test. N.S., non-significant (P > 0.05). * P < 0.05. ** P < 0.01. *** P < 0.001.


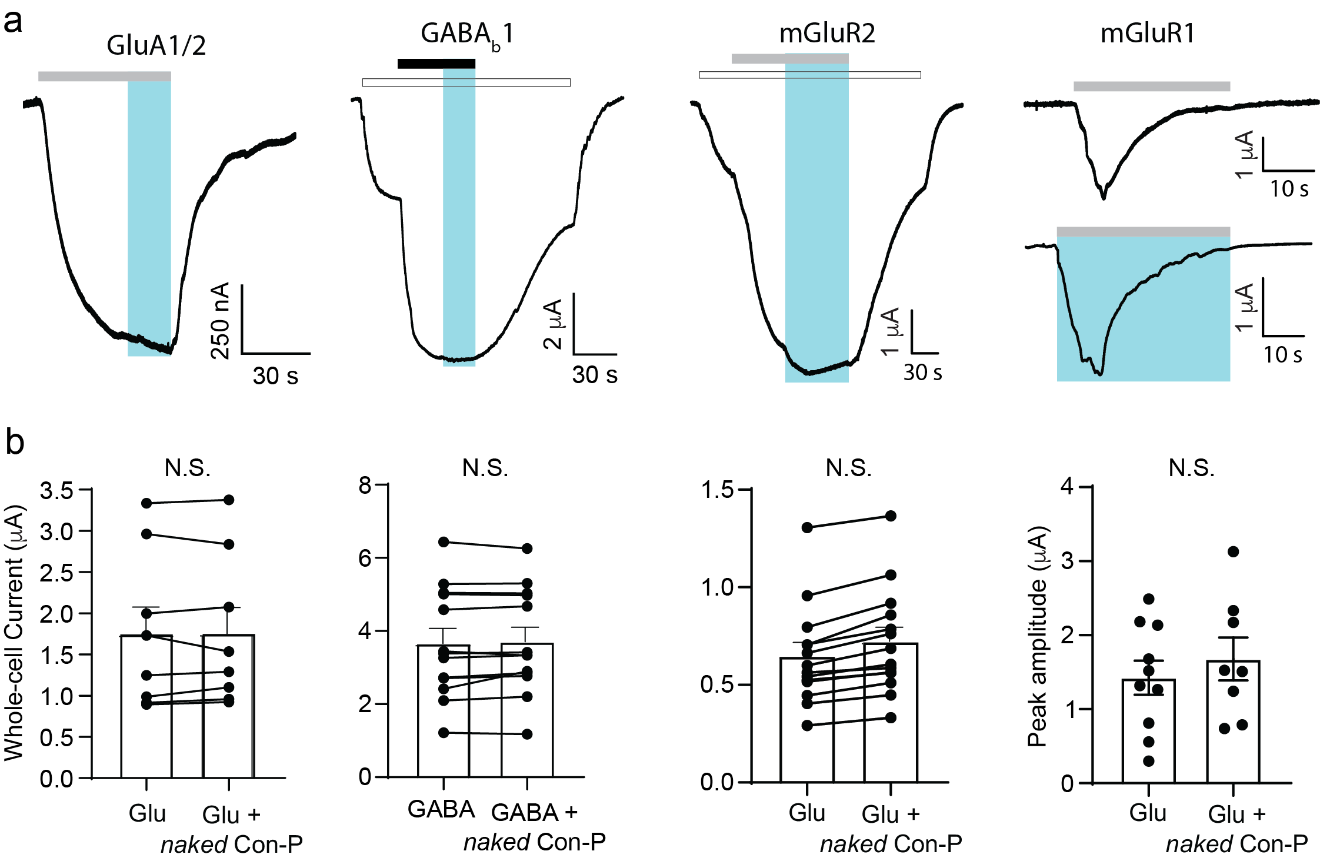


**Supplementary Figure 6. *Naked* Con-P does not affect the activity of non-NMDA receptors. a-b.** Representative recordings from *Xenopus* oocytes expressing AMPARs (GluA1/2; activated by 5 μM glutamate- grey bars), GABA_B_-receptors (activated by 10 μM GABA, grey), mGluR2- or mGluR1-receptors (activated by 10 μM glutamate, grey) and responses to 50 μM *naked* Con-P (cyan). The metabotropic GABA_B_- and mGluR2-receptors were co-expressed with the G-protein inwardly rectifying K^+^-channel (GIRK1/2) and responses (currents) demonstrate inward K^+^-currents. Inward K^+^-current was induced by a high external K^+^ concentration (hK, white bars)^99^. mGluR1 induces activation of endogenous Ca^2+^-activated chloride channels^100^. **b.** Summary of the effect of *naked* con-P over the various receptors. Statistical significance was tested by paired T-tests, except for mGluR1 expressing oocytes. mGluR1 undergoes very strong and near complete desensitization following a single exposure to glutamate^101^. Thus, peak glutamate responses were compared between two groups of oocytes (Glu vs. Glu + *naked* Con-P) by T-test. N.S., non-significant.


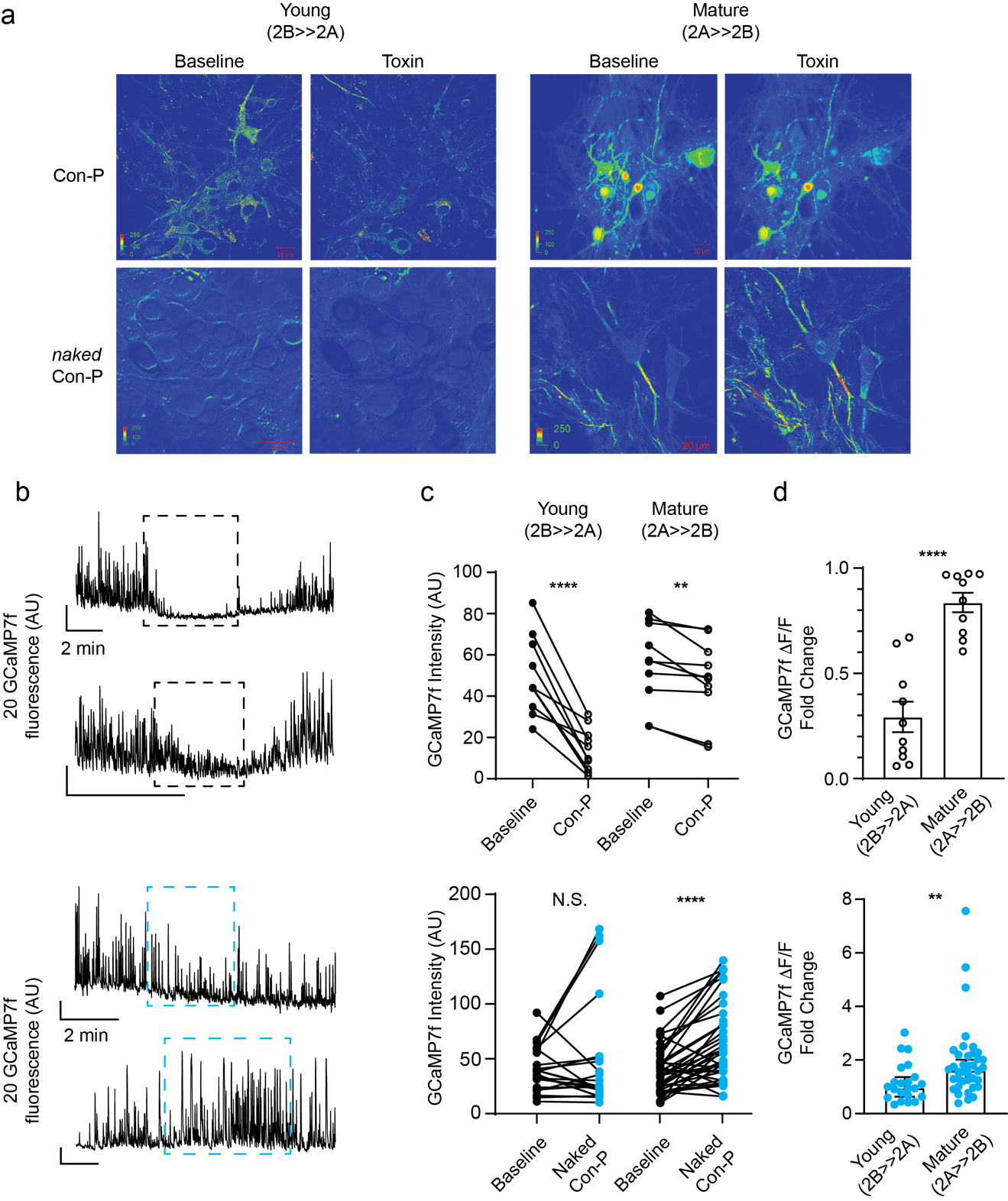


**Supplementary figure 7. Con-P and *naked* Con-P differentially modulate dendritic calcium activity in hippocampal neurons. a.** Confocal images (heat map) of young (9 DIV, left) or mature (16 DIV, right) hippocampal neuron expressing GCaMP7f, before (baseline) and after exposure to 20 μM Con-P or *naked* Con-P (Toxin). **b.** Representative traces of spontaneous Ca^2+^-activity following application of Con-P (top traces, black dashed region) and summaries in (**c, d**- top panels), compared to application of *naked* Con-P (bottom traces, cyan dashed regions), and summaries in (**c, d**- bottom panels). Summaries of GCaMP7f fluorescence intensity (**c**) or fold-change (**d**) before and after toxin application are shown. Statistical significance of repeated measures in (**c**) were tested by two-way ANOVA with *post hoc* Tukey test, whereas (**d**) by T-test. N.S, non-significant. ** P < 0.01, *** P < 0.001.


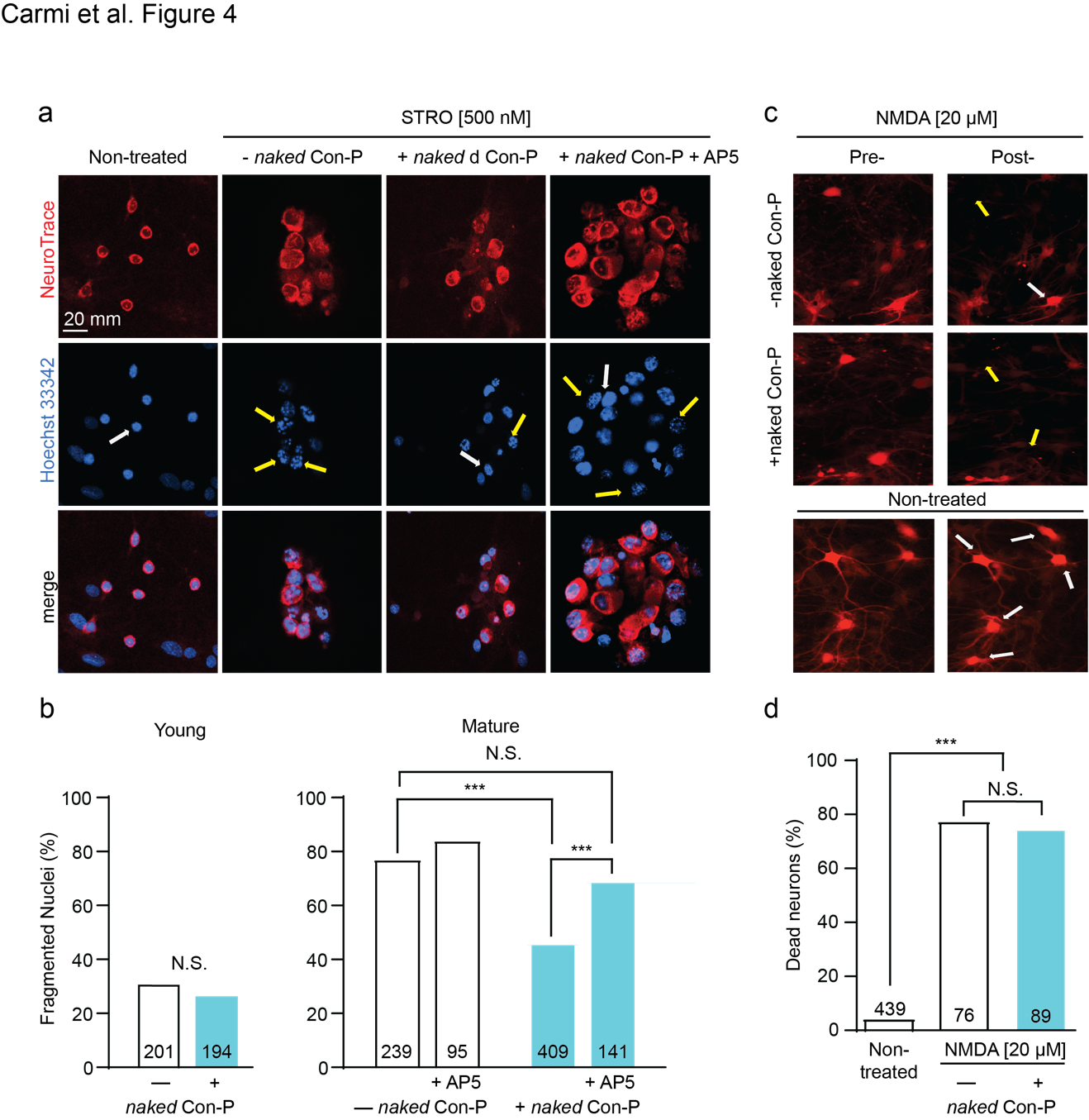


**Supplementary figure 8. *Naked* Con-P proffers neuroprotection to hippocampal neurons exposed to staurosporine, but not NMDA. a.** Confocal images of mature hippocampal neurons exposed to staurosporine (STRO, 500 nM), with (+) or without (-) 24 hrs preincubation with 20 μM *naked* Con-P, compared to naïve (non-treated) cells, and compared to cells that were supplemented with the NMDAR blocker AP5. Neurons were identified based on NeuroTrace-staining (red), and nuclei were examined following Hoechst 33342-staining (blue). White and yellow arrows indicate nuclei of healthy and apoptotic neurons, respectively. **b.** Summary of the fractions of fragmented nuclei from all identified young (left) or mature (right) neurons. Mature neurons treated by *naked* Con-P are neuroprotected, whereas AP5 negates this effect. Statistical significance was tested via Pearson’s Chi-squared test with Yate’s correction (**methods**). **c.** Images of live mature hippocampal neurons virally-expressing tdTomato (red). Neurons were preincubated (24 hrs earlier) with 20 μM *naked* Con-P and imaged in an incubator-based microscope (Incucyte) immediately (left) or 48 hrs (right) after exposure to 20 μM NMDA for 20 minutes, compared to naïve cells (non-treated; bottom). White and Yellow arrows indicate locations of neurons that remained intact or underwent morphological deterioration and clearance, respectively. **d.** Summary of survivability following 48 hours, of non-treated or following NMDA insult. The number of analyzed neurons is indicated within bars. Statistical significance was tested via Pearson’s Chi-squared test. N.S., non-significant; ***, P< 0.001.


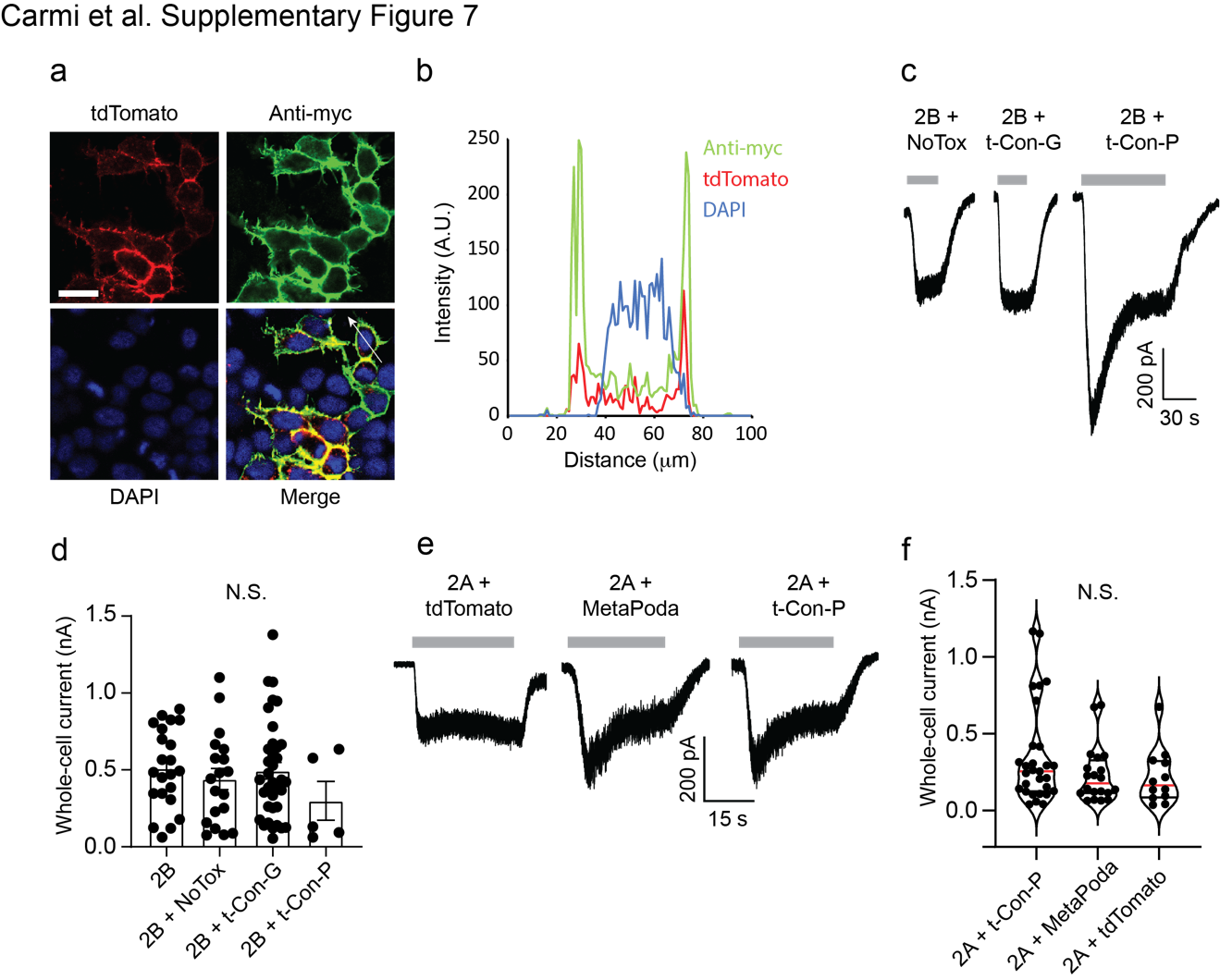


**Supplementary Figure 9. Membrane-tethered Cons fail to modulate NMDARs. a.** Confocal images of HEK293T cells expressing GluN2B-containing receptors and t-Con-G coupled to TdTomato (red). Cells were fixed, immunostained against the Myc-epitope (green) and nuclei stained by DAPI (blue). Scale bar = 50 mm. White arrow indicates trajectory for fluorescent profile analysis in (**b**). Fluorescent profile analysis shows co-localization of t-Con-G and Anti-Myc GFP at membrane of cell. **c.** Whole-cell recordings of HEK293T cells co-expressing GluN2B-containing receptors (2B) and t-con-G or t-Con-P compared to control cells (NoTox) in which the same membrane t-clone was expressed lacking the toxin sequence. Grey bars indicate application of 5 μM glycine and glutamate. **d.** Summary of data shown in (**c**). Statistical significance was tested by One Way ANOVA and *post hoc* Tukey test. **e.** Whole-cell recordings of HEK293T cells co-expressing GluN2A-containing receptors along with t-Con-P (2A + t-Con-P), a control membrane-tethered HeteropodaToxin2 (2A + MetaPoda) or the control tdTomato cytosolic protein (2A + tdTomato). Grey bars indicate application of 5 μM glycine and glutamate. **f.** Summary of data shown in (**e**). Statistical significance tested by Kruskal-Wallis test and *post hoc* Dunn’s multiple comparisons test. N.S., non-significant.


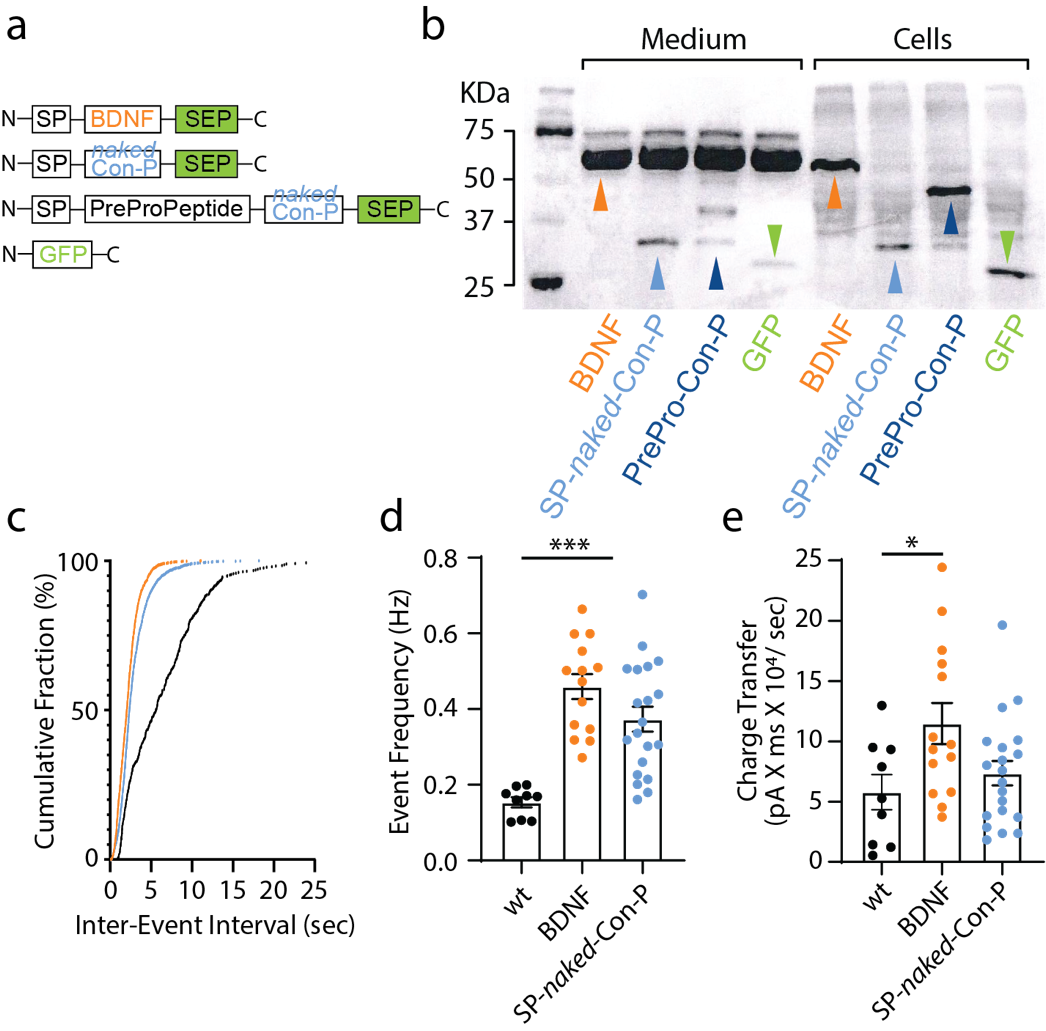


**Supplementary Figure 10. Optimization of secretion of *naked* Con-P in differentiated PC-12 cells. a.** Illustration of the various secreted clones (top to bottom): full length BDNF clone tagged with a pH-sensitive GFP (supereclyptic pHluorin, SEP) including its native signal peptide (SP); a *naked* Con-P tagged with SEP with the SP from BDNF; a *naked* Con-P-SEP clone with SP preceded by a pre-processed sequence of BDNF (PreProPeptide); a non-secreted GFP clone. **b.** Analysis of expression and secretion of the clones. Western blot analysis (immunoblot against GFP) shows presence of peptides in the extracellular medium or cell extracts (cells) of differentiated PC-12 cells transfected with the different clones. Arrowheads indicate BDNF-SEP (orange), SP-naked-Con-P-SEP (cyan), SP-PreProPep-Con-P-SEP (blue) or GFP (green). The inclusion of SP from BDNF in the *naked* Con-P clone (SP-naked-Con-P-SEP, cyan) produces the highest amounts of secreted protein in the medium. Pre-processed BDNF within cells is a larger than the secreted form. **c-e.** Secreted *naked* Con-P has a BDNF-like effect over neurons. Pooled cumulative distribution of sEPSC_NMDAR_ inter-event intervals for naïve (*wt*, black trace) neurons compared to BDNF- or secreted *naked* Con-P-expressing neurons (data from *wt* and secreted *naked* Con-P taken from Fig. 5) (**c**); sEPSC_NMDAR_’s frequency (data from *wt* and secreted *naked* Con-P taken from Fig. 5) (**d**); or charge transfer (**e**) are shown. Statistical significance tested by one-way ANOVA followed by *post hoc* Tukey analysis. *, P < 0.05; ***, P< 0.001.
